# Low-CO_2_-inducible bestrophins outside the pyrenoid sustain high photosynthetic efficacy in diatoms

**DOI:** 10.1093/plphys/kiae137

**Published:** 2024-03-13

**Authors:** Minori Nigishi, Ginga Shimakawa, Kansei Yamagishi, Ryosuke Amano, Shun Ito, Yoshinori Tsuji, Chikako Nagasato, Yusuke Matsuda

**Affiliations:** Department of Bioscience, School of Biological and Environmental Sciences, Kwansei Gakuin University, Sanda, Hyogo 669-1330, Japan; Department of Bioscience, School of Biological and Environmental Sciences, Kwansei Gakuin University, Sanda, Hyogo 669-1330, Japan; Department of Bioscience, School of Biological and Environmental Sciences, Kwansei Gakuin University, Sanda, Hyogo 669-1330, Japan; Department of Bioscience, School of Biological and Environmental Sciences, Kwansei Gakuin University, Sanda, Hyogo 669-1330, Japan; Department of Bioscience, School of Biological and Environmental Sciences, Kwansei Gakuin University, Sanda, Hyogo 669-1330, Japan; Department of Bioscience, School of Biological and Environmental Sciences, Kwansei Gakuin University, Sanda, Hyogo 669-1330, Japan; Graduate School of Biostudies, Kyoto University, Kyoto 606-8502, Japan; Field Science Center for Northern Biosphere, Muroran Marine Station, Hokkaido University, Muroran 051-0013, Japan; Department of Bioscience, School of Biological and Environmental Sciences, Kwansei Gakuin University, Sanda, Hyogo 669-1330, Japan

## Abstract

Anion transporters sustain a variety of physiological states in cells. Bestrophins (BSTs) belong to a Cl^−^ and/or HCO_3_^−^ transporter family conserved in bacteria, animals, algae, and plants. Recently, putative BSTs were found in the green alga *Chlamydomonas reinhardtii*, where they are upregulated under low CO_2_ (LC) conditions and play an essential role in the CO_2_-concentrating mechanism (CCM). The putative BST orthologs are also conserved in diatoms, secondary endosymbiotic algae harboring red-type plastids, but their physiological functions are unknown. Here, we characterized the subcellular localization and expression profile of BSTs in the marine diatoms *Phaeodactylum tricornutum* (PtBST1 to 4) and *Thalassiosira pseudonana* (TpBST1 and 2). PtBST1, PtBST2, and PtBST4 were localized at the stroma thylakoid membrane outside of the pyrenoid, and PtBST3 was localized in the pyrenoid. Contrarily, TpBST1 and TpBST2 were both localized in the pyrenoid. These BST proteins accumulated in cells grown in LC but not in 1% CO_2_ (high CO_2_ [HC]). To assess the physiological functions, we generated knockout mutants for the *PtBST1* gene by genome editing. The lack of PtBST1 decreased photosynthetic affinity for dissolved inorganic carbon to the level comparable with the HC-grown wild type. Furthermore, non-photochemical quenching in LC-grown cells was 1.5 to 2.0 times higher in the mutants than in the wild type. These data suggest that HCO_3_^−^ transport at the stroma thylakoid membranes by PtBST1 is a critical part of the CO_2_-evolving machinery of the pyrenoid in the fully induced CCM and that PtBST1 may modulate photoprotection under CO_2_-limited environments in *P. tricornutum*.

## Teaser

Bestrophin transports HCO_3_^−^ at stroma thylakoid membranes, maintaining the CO_2_-evolving machinery in the pyrenoid of marine diatoms under low CO_2_.

## Introduction

Marine diatoms are a diverse class of marine phytoplankton that is widely spread and responsible for nearly half of oceanic primary production (Falkowski et al. 1998). The diatom lineage arose due to a secondary endosymbiotic event, with diatom cells harboring chloroplasts originally derived from red algae. As is typical in secondary endosymbionts, the diatom chloroplast is composed of 4-layered membranes. The outermost membrane is termed the chloroplast endoplasmic reticulum. The second outer membrane, termed the periplastidal membrane, was derived from the plasma membrane of red algae. The 2 innermost membranes are termed the chloroplast envelopes. Between the periplastidal membrane and outer chloroplast envelope, there is a remnant space left over from the cytosol of the ancestral red algal called the periplastidal compartment (Flori et al. 2016). Within the diatom stroma, there is a triple-layered thylakoid membrane called the girdle lamella with the layered stroma thylakoid at its interior. In the middle of the stroma, there is a phase-separated proteinaceous body called the pyrenoid (Bedoshvili et al. 2009). To sustain high efficacy CO_2_ fixation under seawater conditions where the CO_2_ concentration is low (∼10 *µ*M) but HCO_3_^−^ is predominant (>2 mM), diatoms actively take up external HCO_3_^−^ and/or facilitate diffusive entry of CO_2_ dehydrated from external HCO_3_^−^ by carbonic anhydrases (CAs) expressed in the periplasm (Nakajima et al. 2013; Tsuji et al. 2017). Dissolved inorganic carbon (DIC) taken up from seawater is presumably further mobilized into the chloroplast by an active HCO_3_^−^ transport system on the chloroplast membranes (Matsuda et al. 2017). Finally, DIC in the stroma is converted into CO_2_ at the pyrenoid where the ribulose 1,5-bisphosphate carboxylase/oxygenase (Rubisco) enzyme is condensed (Tsuji et al. 2017). This overall system is termed the biophysical CO_2_-concentrating mechanism (CCM).

The pyrenoid is surrounded by layers of stroma thylakoid membranes and the outermost girdle lamella where the photosynthetic electron transport system produces NADPH and ATP to drive the Calvin cycle. A part of thylakoid membranes traverses through the pyrenoid matrix, which is denoted as the pyrenoid-penetrating thylakoid (PPT) membrane. In the pennate diatom *Phaeodactylum tricornutum*, the θ-type CA, Ptθ-CA1, occurs at the lumen of PPT membranes where light-driven acidification accelerates the dehydration of HCO_3_^−^ to evolve CO_2_ (Kikutani et al. 2016). Similar machinery appears to occur also in the centric diatom *Thalassiosira pseudonana*, because the PPT lumenal θ-type CA was recently identified (Nawaly, Tanaka, et al. 2023). The CA-containing thylakoid membrane at the pyrenoid core appears to be the fundamental structure of the CO_2_-evolving machinery conserved in marine diatoms, whereas the strategies to acquire HCO_3_^−^ from seawater are likely diverse among diatoms. One of the critical missing links to understand the diatom CCM is the system to facilitate influx of HCO_3_^−^ across the thylakoid membranes and into the pyrenoid.

The CO_2_-evolving machinery coupled with lumenal CA also occurs in the pyrenoid of the green alga *Chlamydomonas reinhardtii*, strongly suggesting the occurrence of convergent evolution has taken place across distant taxa of algae. Given the steady and continual decrease in atmospheric CO_2_ concentration over Earth's history, such a system would have been tightly selected to optimize the use of light and CO_2_ in the aquatic environment. It is recently reported that the bestrophin (BST) putative paralogs BST1 to 3 localized in the thylakoid membranes transport HCO_3_^−^ to support the CO_2_-evolving reaction by the α-type CA, CAH3, in the lumen of the pyrenoid-invaginating thylakoid membranes (termed as the pyrenoid tubule) in *C. reinhardtii* (Mukherjee et al. 2019), most probably as a part of the CO_2_-evolving machinery in *C. reinhardtii*. Transcripts of *BST1 to 3* in *C. reinhardtii* were highly accumulated in cells grown under atmospheric CO_2_ (low CO_2_ [LC]) but were found only in trace amounts in 5% CO_2_ (high CO_2_ [HC])-grown cells, clearly indicating the LC-inducible nature of these BSTs (Mukherjee et al. 2019). *BST* expression in *C. reinhardtii* probably occurs in concert with the structural dynamics of the pyrenoid in response to CO_2_. Such structural dynamics with controls of *BST* expression is required to play a specific role in the CCM under CO_2_ limitation. Even though the function of these BSTs seemed tightly related to the function of the pyrenoid tubules, BSTs in LC-grown *C. reinhardtii* were likely localized not just in the pyrenoid tubules but also in other areas of the thylakoid membranes (Mukherjee et al. 2019).

Bicarbonate is an anion and thus could counteract the H^+^-derived thylakoid membrane potential. Conversely, a high activity of HCO_3_^−^ dehydration catalyzed by thylakoid lumenal CA results in the consumption of H^+^, potentially dissipating the proton gradient across the membranes (ΔpH). These potential effects of the introduction of HCO_3_^−^ into the thylakoid lumen could impose a substantial impact on the function of the photosynthetic electron transport. The problem of the effect of HCO_3_^−^ introduction into the lumen of the spatially and functionally diverse thylakoid membrane system is still an open question in *C. reinhardtii*. Diatoms also possess putative BST sequences in their genomes, but the function of these putative candidates, localizations, or expressional controls is not yet totally known.

Here, we searched putative BST genes and found 4 and 2 isoforms in *P. tricornutum* and *T. pseudonana*, respectively. These putative BSTs were localized in the different chloroplast areas and highly expressed in response to CO_2_ limitation in regulation at either transcriptional or posttranscriptional processes. The most abundant isoform in *P. tricornutum*, denoted as *PtBST1*, was targeted by a highly specific genome editing (CRISPR/Cas9 nickase) to generate knockout mutants. The photosynthetic phenotypes of the mutants suggest that PtBST1 functions as the HCO_3_^−^ transporter in the LC-inducible CCM in *P. tricornutum* and further might modulate photoprotective mechanisms in relation to the utilization of H^+^ in the thylakoid lumen.

## Results

### Screening of BST orthologs in marine diatoms

BST is often defined as a Ca^2+^-activated anion transporter (Yang et al. 2014) and its sequences were clustered into several large protein families in a variety of organisms, including bacteria, animals, and plants. Indeed, a BLAST search for Chlamydomonas BST1 to 3 sequences found multiple BST homologs in each species in diverse photosynthetic organisms, including cyanobacteria, green algae, red algae, land plants, and secondary algae (Supplementary Fig. S1). Among these homologs, the *C. reinhardtii* BST1 to 3 (Mukherjee et al. 2019) form a large clade including land plants, cryptomonads, haptophytes, and diatoms. In this study, we defined the orthologs categorized into this clade as “BST” for the candidates of HCO_3_^−^ transporter in the thylakoid membranes.

**Figure 1.**
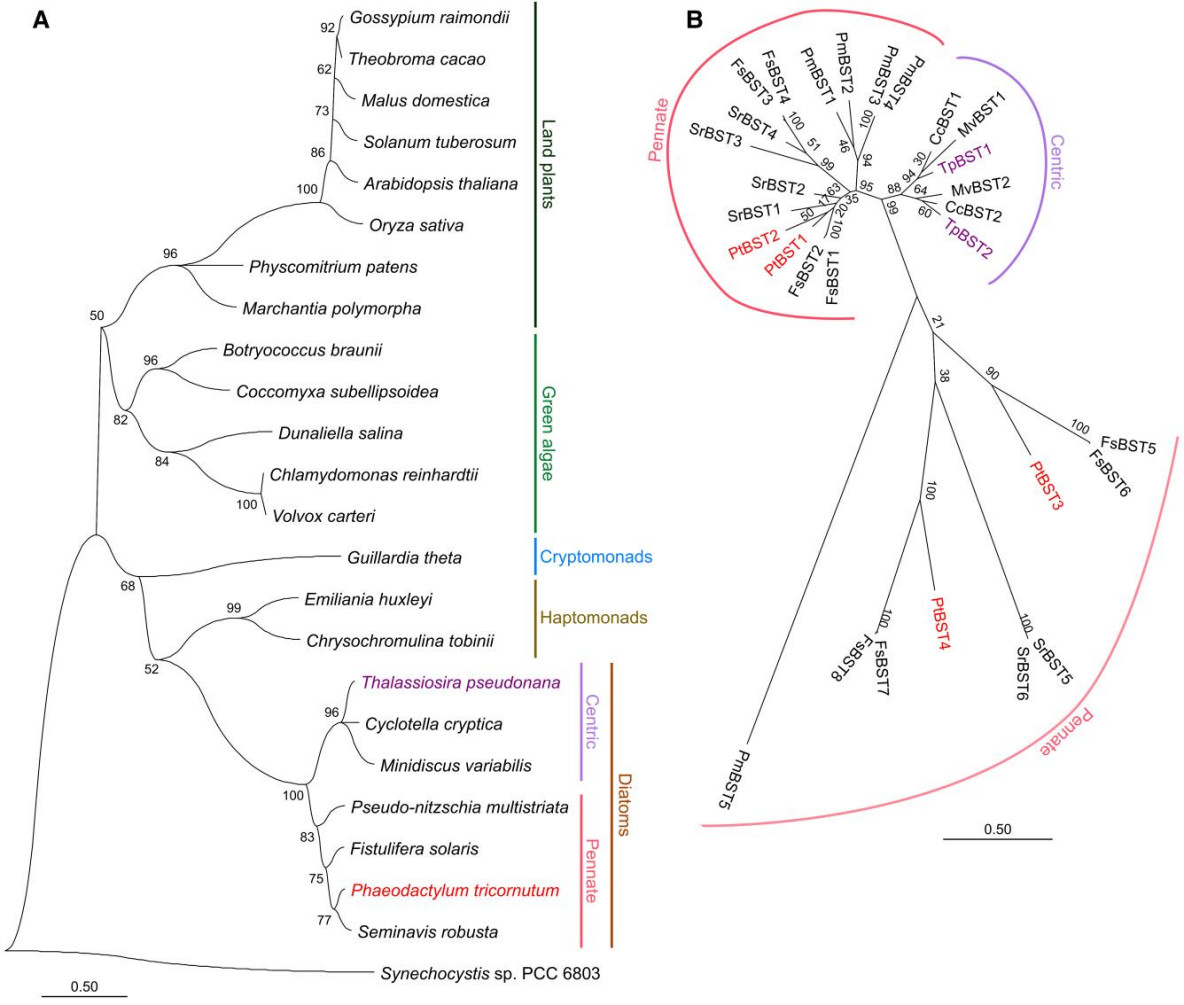
Phylogeny of BST orthologs in photosynthetic organisms. **A)** Evolutionary relationship inferred by the maximum likelihood method with Le and Gascuel (2008) model. One BST ortholog per species showing the highest similarity to PtBST1 was used for the phylogenetic analysis. The cyanobacterium *Synechocystis* sp. PCC 6803 is an outgroup. Initial trees for the heuristic search were obtained automatically by applying Neighbor-Join and BioNJ algorithms to a matrix of pairwise distances estimated using the JTT model (see Materials and methods for the details). **B)** Similarity of BST isoforms in 7 diatom species. Species names were abbreviated as follows: *P. tricornutum* (Pt), *T. pseudonana* (Tp), *Pseudo-nitzschia multistriata* (Pm), *Fistulifera solaris* (Fs), *Seminavis robusta* (Sr), *Cyclotella cryptica* (Cc), and *Minidiscus variabilis* (Mv).

BST orthologs are widely found in green algae, land plants, and secondary algae such as haptophytes and diatoms (Fig. 1A). Diatom BSTs further constituted a specific subclade that is apart from the subclade of the green linage. Meanwhile, cyanobacteria, red algae, and some secondary algae do not possess the BST orthologs. Further, the BST orthologs could not be recognized in the Prasinophyceae algae *Ostreococcus* (Supplementary Fig. S1), suggesting that the BST in green lineage might have been acquired in the common ancestor of streptophytes and chlorophytes and thereafter inherited by plants. It is likely that BST in secondary algae, such as cryptomonads, haptophytes, and diatoms, might be derived via a past temporal endosymbiotic association (cryptic endosymbiosis) with a green alga prior to the acquisition of present red-type chloroplast (Moustafa et al. 2009), whereas the BST homologs had been lost in some secondary endosymbiotic algae. More than 2 BST isoforms were identified in 7 diatom species whose genome information is presently available (Fig. 1B): pennate diatoms encode more than 3 BSTs, while centric diatoms harbor 2 BST isoforms. In *P. tricornutum*, PtBST1 (Phatr3_J46336) and PtBST2 (Phatr3_J26635) share high similarity (approximately 66%) and formed a pennate-specific clade, whereas PtBST3 (Phatr3_J46366) and PtBST4 (Phatr3_J46360) were in a separate group (Fig. 1B). *T. pseudonana* has 2 BST putative paralogs (TpBST1, THAPSDRAFT_4819; and TpBST2, THAPSDRAFT_4820), which show approximately 66% similarity and are categorized into the centric-specific clade adjacent to the pennate-specific group that includes PtBST1 and 2 (Fig. 1B).

One more BST isoform (Phatr3_J46365) was suggested by the database to occur in *P. tricornutum* showing 73% similarity to PtBST3, which could be denoted as PtBST5. Since the coding sequence of *PtBST5* is possibly derived from a splice variant or other allele of PtBST3, PtBST5 was excluded from the main subject in this study. Nevertheless, we note that the C-terminal extension region of PtBST3 is specifically lost in PtBST5, although these 2 BST isoforms show almost the same amino-acid sequences (Supplementary Fig. S2).

### Subcellular localization of diatom BST isoforms

Based on the coding sequences from available databases, all BST isoforms identified above, except for PtBST4 and TpBST1, were predicted to have the chloroplast transit peptide (Gruber et al. 2007). However, in this study, a 5ʹ-RACE analysis of PtBST4 and TpBST1 identified an upstream start codon that was missing in the database, and the chloroplast transit peptide was predicted in the recently obtained sequences of PtBST4 and TpBST1. Overall, all BST isoforms in *P. tricornutum* and *T. pseudonana* were predicted to be localized in chloroplasts (Supplementary Fig. S3).

**Figure 2.**
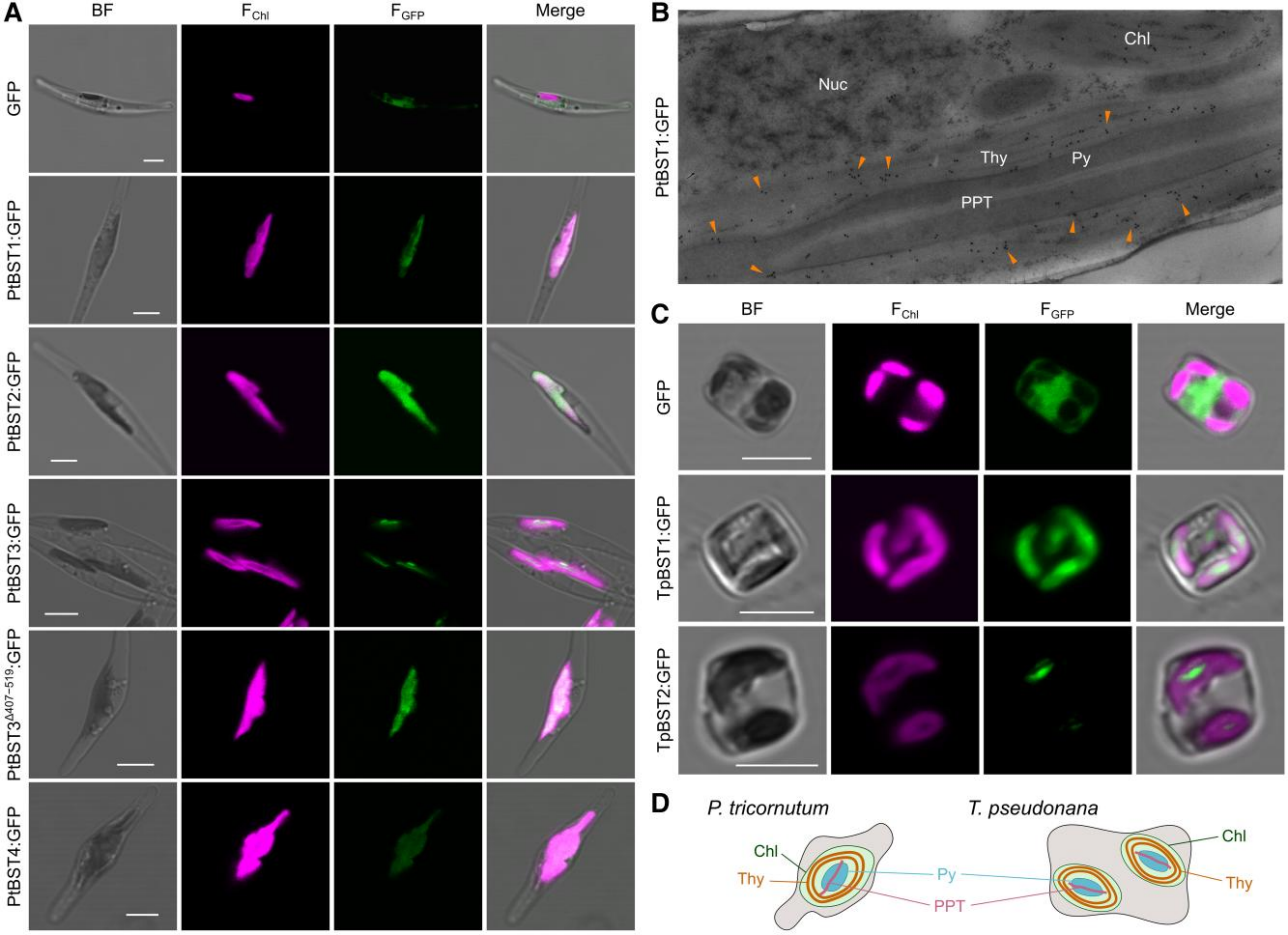
Subcellular localization of BST isoforms in *P. tricornutum***A, B)** and *T. pseudonana***C)**. **A, C)** Confocal images of bright field (BF), chlorophyll autofluorescence (*F*_Chl_), green fluorescence derived from GFP tagged to BST (*F*_GFP_), and all merged. White bars indicate 5 *µ*m. **B)** Immunoelectron microscopy with a specific antibody to GFP in the *P. tricornutum* transformant that expresses GFP-tagged PtBST1. **D)** Simple illustrations of *P. tricornutum* and *T. pseudonana* cells. Nuc, nuclei; Chl, chloroplasts; Thy, stroma thylakoid membranes; Py, pyrenoid; PPT, pyrenoid-penetrating thylakoid membranes.

To determine the specific localization in chloroplasts, we generated the transformants that express GFP-fused BST isoforms in *P. tricornutum* and *T. pseudonana*. The green fluorescence derived from PtBST1:GFP, PtBST2:GFP, and PtBST4:GFP was detected across the whole chloroplast (Fig. 2A). The localization of PtBST1:GFP was further analyzed by electron microscopy with a GFP-specific antibody (Fig. 2B), clearly showing its localization within the thylakoid membrane outside of the pyrenoid. These data suggest that PtBST1, PtBST2, and PtBST4 are localized mainly in the stroma thylakoid membrane outside of the pyrenoid (Fig. 2D). Meanwhile, the green fluorescence derived from GFP-fused PtBST3 was recognized specifically in a center part of chloroplasts where chlorophyll fluorescence was absent (Fig. 2A), an area most probably of the pyrenoid, strongly suggesting that PtBST3 occurs in PPT membrane (Fig. 2D). To reveal a sequence motif that localizes PtBST3 to the pyrenoid, we further analyzed the subcellular localization of a truncated PtBST3 lacking in the C-terminal extension sequence (G407–P519) that specifically occurs in PtBST3 but not in PtBST5 (Supplementary Fig. S2) by expressing as the GFP fusion protein. The truncated PtBST3^Δ407–519^:GFP altered its location, and the GFP signal was detected throughout the chloroplast in contrast to PtBST3:GFP, suggesting that the C-terminal region of PtBST3 contains the key targeting motif to be sorted to the pyrenoid in *P. tricornutum* (Fig. 2A).

In *T. pseudonana*, both TpBST1 and TpBST2 were found to be localized in the thylakoid membranes within or around the pyrenoid according to the GFP signal of respective GFP fusion proteins (Fig. 2C), which is consistent with the latest data of TpBST2 localization by Nam et al. (2022). Compared with TpBST2, the green fluorescence of TpBST1:GFP was detected broadly in the chloroplast (Fig. 2C). TpBST1 thus might be localized in both stroma thylakoid and PPT membranes (Fig. 2D).

### Responses of the expression of diatom BSTs to changing CO_2_ concentrations

It has been reported that the CCM components are highly expressed under CO_2_ limitation in most cases at transcript levels in marine diatoms (Ohno et al. 2012; Nakajima et al. 2013; Hennon et al. 2015). Here, we found “TGACGT/C” motif, which is a target of a basic zipper (bZIP) 11 transcription factor in *P. tricornutum* (Ohno et al. 2012), in the upstream genome sequence (∼300 bp from the start codon; 2 and 4 motifs at the sense and antisense strands, respectively) of *PtBST1*, while those of *PtBST2*, *PtBST3*, and *PtBST4* respectively contain 4, 1, and 0 motifs only at the one side strand (Supplementary Fig. S4). The motif set similar to that in *PtBST1* has been previously characterized in the promoter region for the LC-inducible β-CA1 gene, *PtCA1*, in *P. tricornutum* and defined as the CO_2_-cAMP-responsive elements (Ohno et al. 2012). Therefore, *PtBST1* was expected to be repressed at 1% CO_2_ (HC) concentration via the cAMP-dependent signaling pathway. Additionally, in *T. pseudonana*, both TpBST1 and TpBST2 have been found to be upregulated under CO_2_ limitation in a proteomic analysis (Kustka et al. 2014).

**Figure 3.**
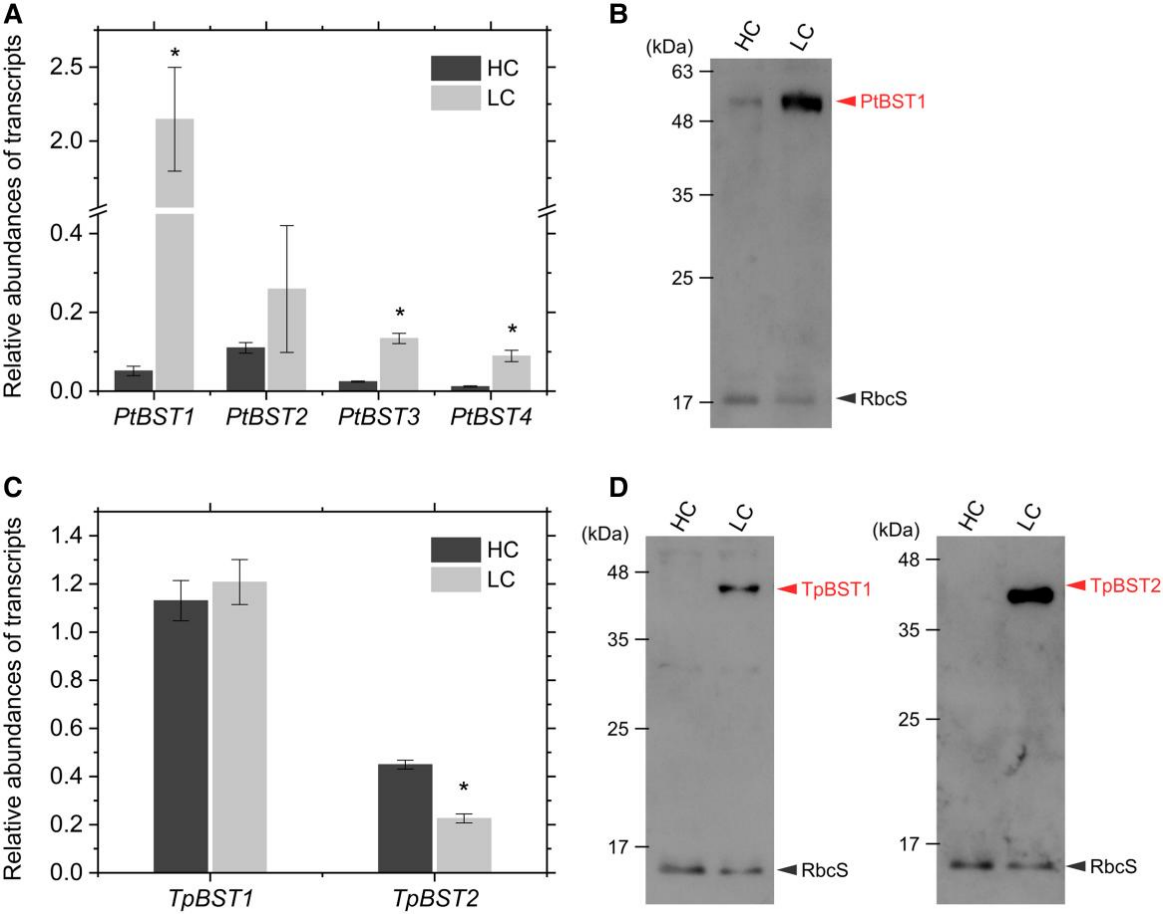
Expression of *BST* isoforms under air-level (LC) and 1% (HC) CO_2_ in *P. tricornutum* and *T. pseudonana*. **A, C)** Relative abundances of transcripts estimated by reverse transcription qPCR with *Actin1* as the reference gene. Data are shown as the mean ± Sd (*n* = 3, biological replicates). Asterisks indicate statistically significant differences (*P* < 0.05) between HC and LC as per Student's *t*-test. **B, D)** Accumulation of PtBST1, TpBST1, and TpBST2 proteins estimated by immunoblotting with each specific antibody. rbcS protein was used as the loading control. Representative data of 3 independent experiments are shown.

Here, we examined the expression levels of 4 and 2 BST isoforms in *P. tricornutum* and *T. pseudonana*, respectively. Compared with the HC condition, cells grown under LC condition highly accumulated *PtBST1 to 4* transcripts (Fig. 3A). In particular, the transcript level of *PtBST1* was higher (10 to 20 times) than those of other *PtBSTs*, and the induction ratio of *PtBST1* transcript in LC over HC was more than 40 times (Fig. 3A), strongly suggesting that PtBST1 is the most abundant BST isoform in *P. tricornutum*. The accumulation of PtBST1 at the protein level was also investigated by immunoblotting with newly generated anti-PtBST1 antiserum. Consistent with the transcript level, PtBST1 protein was highly expressed in the LC condition, while in HC-grown cell lysate, PtBST1 was found only in trace amounts (Fig. 3B). In *T. pseudonana*, in sharp contrast to *P. tricornutum*, the abundance of the *TpBST1* and *TpBST2* transcripts was not different between HC- and LC-grown cells, and the transcript of *TpBST2* was even lower in the LC condition (Fig. 3C), indicating that the expression of TpBSTs was not controlled at the transcript level. However, interestingly, both TpBSTs isoforms were never detected at the protein level in the cells grown in HC but highly accumulated in LC-grown cells (Fig. 3D). These data strongly suggest that the expression of BSTs in *T. pseudonana* is induced by CO_2_ limitation at the posttranscriptional levels; in other words, translation of these *TpBSTs* transcripts is almost completely suppressed in HC. Given these data, the regulatory mechanisms of BST expression in response to CO_2_ concentrations between these 2 diatom species are totally different.

### Highly specific genome editing of *PtBST1* using CRISPR/Cas9 nickase

In this study, we performed the gene disruptions of *PtBST1* in *P. tricornutum* by the highly specific genome-editing tool CRISPR/Cas9 (D10A) nickase optimized for diatoms (Nawaly et al. 2020). Transformed colonies were respread more than twice on agar plates containing antibiotics to obtain the monoclones. Finally, two lines of independent biallelic knockout mutants were successfully obtained, denoted as ΔPtBST1_1 and ΔPtBST1_2 (Supplementary Fig. S5, A and B). Immunoblotting using the specific PtBST1 antibody indicated that these mutants lacked PtBST1 at the protein level (Supplementary Fig. S5C).

**Figure 4.**
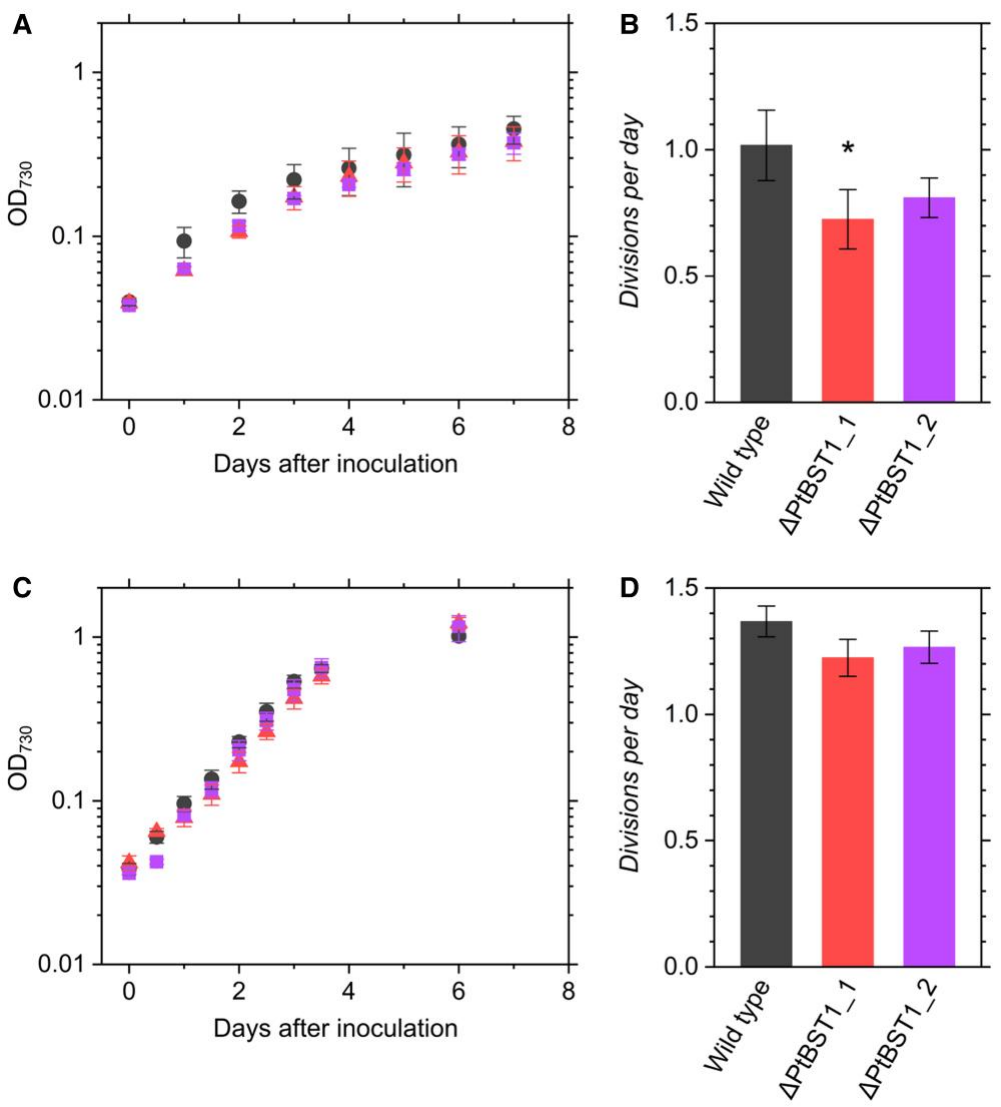
Growth of *P. tricornutum* WT (black circles) and the PtBST1 knockout mutants (ΔPtBST1_1, red triangles; ΔPtBST1_2, purple squares) under LC **(A, B)** or HC **(C, D)** condition. Data are shown as the mean ± Sd (*n* = 3, biological replicates). *Divisions per day* was calculated at the logarithmic growth phase **(B, D)** (see Materials and methods for the details). The asterisk indicates statistically significant difference (*P* < 0.05) between WT and the mutants as per Student's *t*-test.

These knockout mutants, ΔPtBST1_1 and ΔPtBST1_2, showed a trend of slightly retarded growth rate in the LC conditions (Fig. 4A). The growth at the logarithmic phase was evaluated as daily specific doubling rate, the so-called *divisions per day* (Wood et al. 2005). ΔPtBST1_1 mutant showed a significantly lower growth rate compared with the wild type (WT) (Fig. 4B). Meanwhile, there was no difference in the growth between the WT and knockout mutants in HC conditions (Fig. 4, C and D).

### Effects of the lack of PtBST1 on photosynthesis of *P. tricornutum*

**Figure 5.**
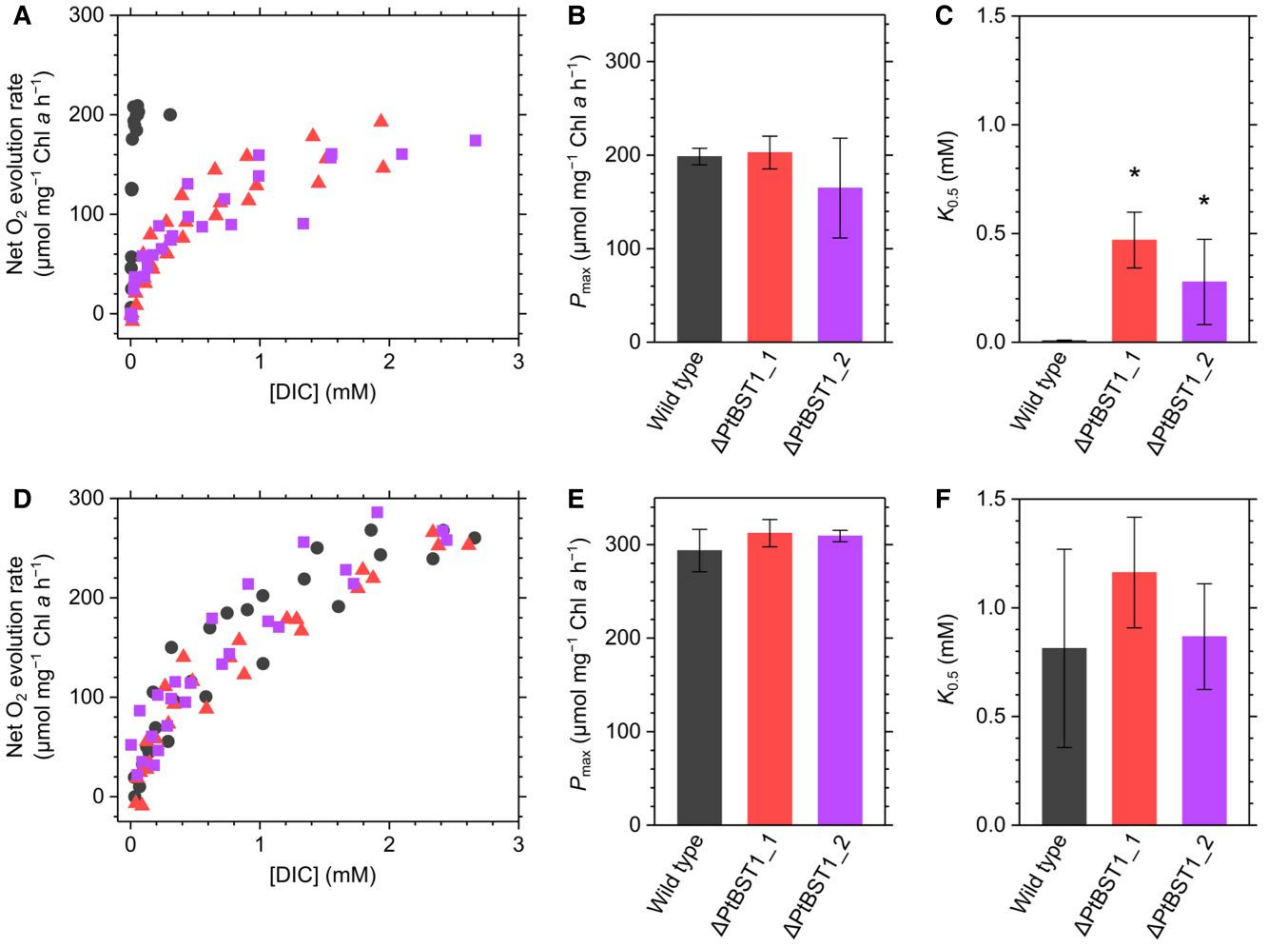
Net O_2_ evolution rate at different concentrations of DIC in *P. tricornutum* WT (black circles) and the PtBST1 knockout mutants (ΔPtBST1_1, red triangles; ΔPtBST1_2, purple squares) grown under LC **(A, B, C)** or HC **(D, E, F)** condition. Measurements were conducted under the illumination with a white actinic light (800 *µ*mol photons m^−2^ s^−1^). Data of 3 independent experiments are all plotted in Fig. 5, A and D, from which the maximum O_2_ evolution rate (*P*_max_; **B, E)** and the DIC concentration giving to a half of *P*_max_ (*K*_0.5_; **C, F)** were calculated to show as the mean ± Sd. Asterisks indicate statistically significant differences (*P* < 0.05) between WT and the mutants as per Student's *t*-test.

We determined the photosynthetic parameters (DIC affinity and maximum rate) by measuring the kinetics of net O_2_ evolution rate over various DIC concentrations, which were quantified with gas chromatography flame ionization detector (GC-FID). Whereas the maximum photosynthetic O_2_ evolution rate (*P*_max_) was not significantly different between the LC-grown WT and LC-grown mutant cells, at around 200 *µ*mol O_2_ mg^−1^ chlorophyll *a* h^−1^, both LC-grown mutants showed a significantly lower photosynthetic DIC affinities as indicated by the *K*_0.5_ value; that is, 0.3 to 0.5 mM for the knockout mutants, while in the LC-grown WT cells, it was about 0.01 mM DIC (Fig. 5, A to C). These kinetics data clearly indicate that, in BST1-KO mutants, the photosynthetic DIC affinity decreased to 1.5% of that of the WT cells. In the cells grown under HC, there was no difference in either parameter between WT and mutant strains, and both *P*_max_ and *K*_0.5_ were higher than in the cells grown under LC (Fig. 5, D to F). Importantly, *K*_0.5_ was higher in the HC-grown cells than in the LC-grown cells even in the absence of PtBST1 (Fig. 5, C and F), suggesting that something other than *PtBST1* repressed CCM factors in WT cells under HC.

**Figure 6.**
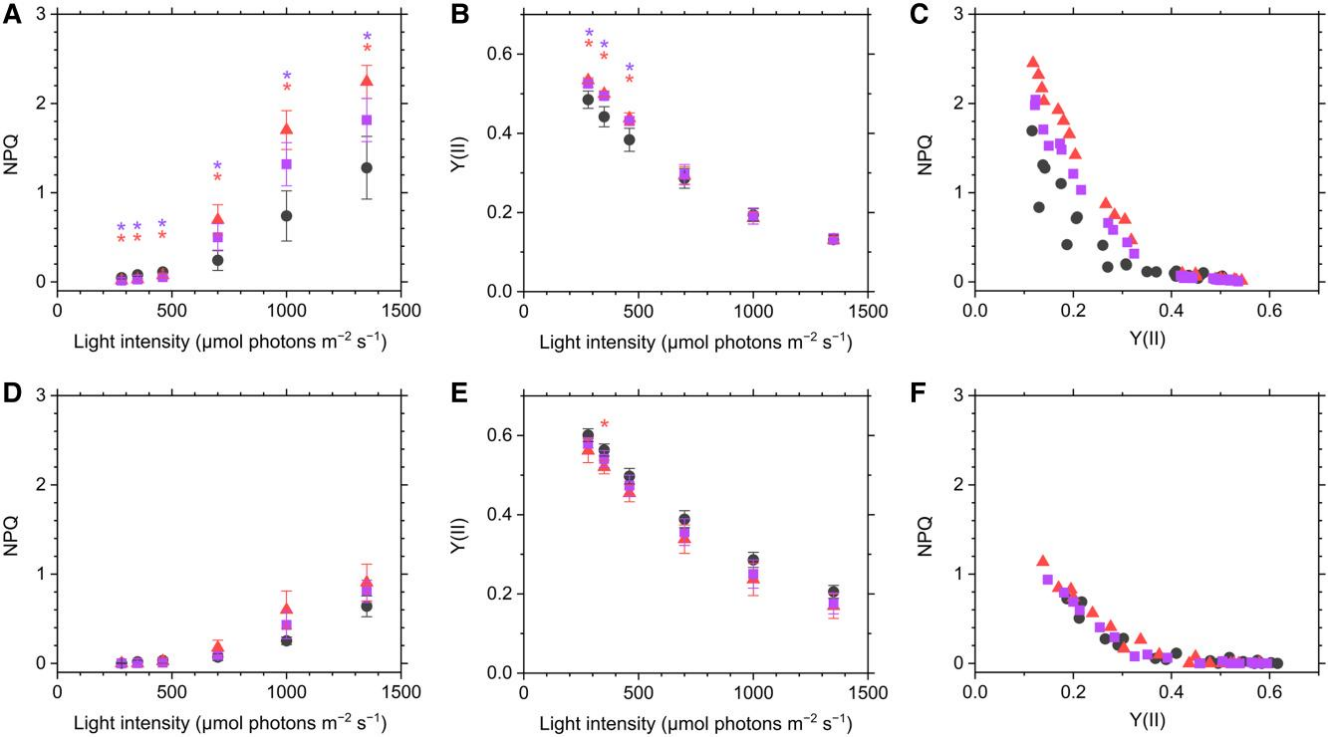
Chlorophyll fluorescence parameters at different light intensities in *P. tricornutum* WT (black circles) and the PtBST1 knockout mutants (ΔPtBST1_1, red triangles; ΔPtBST1_2, purple squares) grown under LC **(A, B, C)** or HC **(D, E, F)** condition. NPQ **(A, D)** and effective quantum yield of PSII, termed as Y(II) **(B, E)**, were calculated. Measurements were conducted in the presence of 10 mM NaHCO_3_. Data are shown as the mean ± Sd (**A to C**: *n* = 4; **D to F**: *n* = 3, biological replicates). NPQ values were plotted against Y(II) in **C)** and **F)**.

Non-photochemical quenching (NPQ) was significantly increased to 1.5 to 2.0 times in both mutants relative to that in the WT cells when the measurement was done under CO_2_-saturating condition for LC-grown cells (Fig. 6A). Effective quantum yield of PSII, termed as Y(II), was slightly higher in the mutants than in the WT at low light intensities (Fig. 6B), but we could not clarify the mechanism for this difference. NPQ and Y(II) are inversely associated during the steady-state photosynthesis under changing actinic light intensity in all strains. Importantly, the mutants always showed higher NPQ versus Y(II) relative to that of WT (Fig. 6C), strongly suggesting that PtBST1 adds some effect to modulate the energy distribution system on PSII. In the HC-grown cells, NPQ was lower in both WT and mutants, compared with the LC-grown cells, and the relationship between NPQ and Y(II) was similar among them (Fig. 6, D to F).

## Discussion

**Figure 7.**
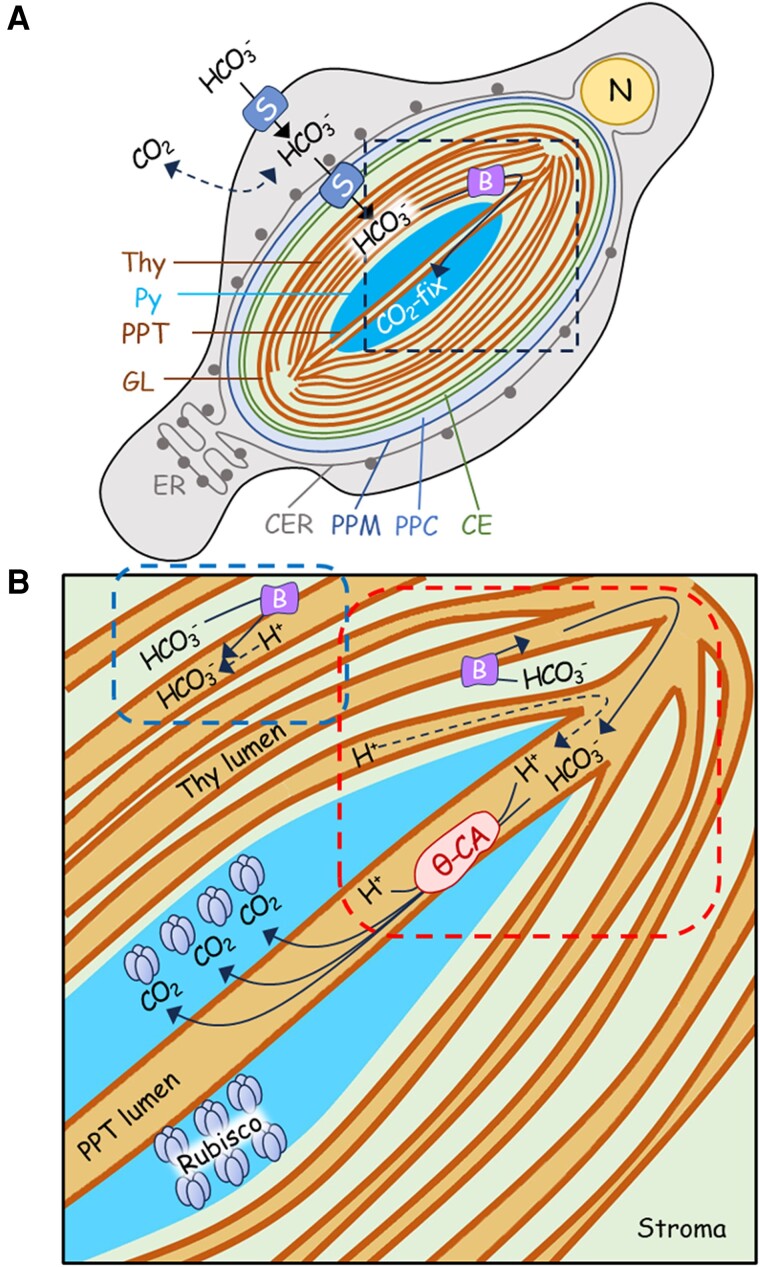
Schematic drawings of the CCM and possible BST functions in the marine diatom *P. tricornutum*. **A)** A whole-cell system of taking up and mobilizing DIC toward CO_2_ fixation by Rubisco at the pyrenoid; **B)** magnified image of dashed box in **A)** indicating the close look of DIC movement around the pyrenoid; HCO_3_^−^ imported into the stroma thylakoid lumen by PtBST1 is mobilized to the lumen area within the pyrenoid and is provided to the CO_2_-evolving machinery in the pyrenoid. Blue box indicates the HCO_3_^−^ interaction with ΔpH and/or H^+^ consumption by spontaneous HCO_3_^−^ dehydration at the stromal thylakoid; red box indicates H^+^-drawn down from the stroma thylakoid by the CO_2_-evolving machinery. N, nuclei; Thy, stroma thylakoid membranes; Py, pyrenoid; PPT, pyrenoid-penetrating thylakoid membranes; GL, girdle lamellae; CER, chloroplast endoplasmic reticulum; PPM, periplastidal membrane; PPC, periplastidal compartment; CE, chloroplast envelope; B, PtBST1; S, solute carrier 4 HCO_3_^−^ transporter.

Aquatic photosynthesis has evolved the biophysical CCM to overcome low CO_2_ availability within the marine environment, with such evolution likely accelerated under the low CO_2_/O_2_ environment after the Carboniferous. Efficient acquisition of abundant external HCO_3_^−^ (ca. 2 mM) results in high accumulation of stroma HCO_3_^−^, which is eventually converted to CO_2_ by the CO_2_-evolving machinery in the pyrenoid that provides an ample flux of CO_2_ substrate to Rubisco condensate in the pyrenoid (He et al. 2023). In the case of marine diatoms, it is known that the PPT lumen is the site and θ-type CA plays a role as the converter in the CO_2_-evolving machinery (Fig. 7; Kikutani et al. 2016; Shimakawa et al. 2023).

The above-mentioned general model, however, contains several unsolved problems, the clarification of which is essential to understand the biophysical CCM in diatoms. Of particular importance, (i) the mechanism to maintain inner chloroplast structure with the pyrenoid and functionally diverse thylakoid membrane, (ii) the system to provide the accumulated stroma HCO_3_^−^ into the CO_2_-evolving machinery in the PPT lumen, and (iii) the mechanism to prevent the efflux of unfixed CO_2_ from the chloroplast are the immediate issues to be solved.

In the present study, we provide strong evidence that BSTs supply HCO_3_^−^ to the CO_2_-evolving machinery in the PPT lumen. In a recent study, we found that a complete defect of Ptθ-CA1 in *P. tricornutum* significantly lowered the DIC affinity for photosynthesis (*K*_0.5_ > 2 mM DIC and photosynthesis only saturated at more than 10 mM DIC) (Shimakawa et al. 2023), indicating that the CO_2_-evolving machinery is essential for photosynthesis not only under CO_2_ limited conditions but also even under CO_2_ enrichment 5 times greater than current atmospheric levels. In support of this, PPT components such as the lumenal Ptθ-CA1 are constitutively maintained regardless of the growth CO_2_ concentrations (Kikutani et al. 2016), while the presence of other components like BSTs is clearly stimulated by LC at either transcript or posttranscriptional levels (Fig. 3, A and B). This indicates that the diatom biophysical CCM is composed of 2 systems: one is the constitutively operating CCM within the diatom pyrenoid that is absolutely required to sustain photosynthesis in seawater by maintaining the pyrenoid with thylakoid membranes harboring CA, and the other is the LC-inducible CCM that highly strengthens the activity of DIC acquisition and the function of the pyrenoid-based CO_2_-evolving machinery. The latter mechanism is sustained by a few LC-inducible CCM factors that, in addition to BSTs, include previously identified proteins such as solute carrier 4 HCO_3_^−^ transporters at the plasma membrane (Nakajima et al. 2013; Nawaly, Matsui, et al. 2023), still unidentified HCO_3_^−^ transporters in the chloroplast membrane system and pyrenoidal CAs (Satoh et al. 2001; Harada and Matsuda 2005).

In diatoms, the mode of operation of their CCM under subatmospheric to very low-level CO_2_ (VLC) is totally unknown, while, in the green alga, *C. reinhardtii*, numerous mutagenesis experiments have indicated that a different type CCM mode operates in VLC as compared with LC. In VLC, *C. reinhardtii* relies more on chloroplast envelope HCO_3_^−^ transporters, while in LC, the CCM primarily utilizes less energy-requiring methods using a CO_2_-leakage barrier, such as LCIB/C complex (putative θ-type CA) from the stroma (Duanmu et al. 2009; Yamano et al. 2010; Fei et al. 2022). Given these examples from green alga, it is very possible that diatoms also operate a further strengthened mode of the CCM under VLC, which would be another interesting topic to be studied. In the present study, we showed that PtBST1 is one of critical components that facilitate the LC-inducible mode of the CCM but is not involved in the constitutively operating CCM in *P. tricornutum* as the photosynthetic DIC affinity of ΔPtBST1 was still much higher than that of the mutants of *P. tricornutum* defective of Ptθ-CA1, which is the mutant lacks in constitutively operating CCM (Shimakawa et al. 2023).

It should be noted that the system by which BST expression is regulated differs quite markedly between the 2 species used in the present study. Especially in *T. pseudonana*, it is clear that the production of the BST protein is strongly regulated at the translational, rather than transcriptional, level in response to the ambient CO_2_ concentrations (Fig. 3). CO_2_ responses of diatom CCM factors have been studied mostly at the transcriptional levels (Ohno et al. 2012; Hennon et al. 2015). So far, there are not many examples of posttranscriptional regulation in diatoms, and thus our data on *T. pseudonana BST* would provide an intriguing sample to study the posttranscriptional controls of protein expression in response to CO_2_. As for the transcriptional controls, it is known that cAMP plays a role as a secondary messenger to transmit a CO_2_ signal to CO_2_-responsive *cis*-element via a bZIP-type transcription factor PtbZIP11 (Harada et al. 2006; Ohno et al. 2012; Hennon et al. 2015), and indeed, our present study revealed that the *PtBST1* gene also possesses a typical tandem invert repeat of the TGACGT/C motif (Supplementary Fig. S4). This suggests that the expression of *PtBST1* gene is regulated in response to CO_2_ concentrations in the same manner to that of *PtCA1* via a cAMP-dependent mechanism (Tanaka et al. 2015). In contrast, the expression levels of *PtBST2*, *PtBST3*, and *PtBST4* might be regulated by different mechanisms from those of *PtBST1* (Fig. 3A) as their promoter sequences only possess multiple TGACGT/C motifs on 1 strand, different from those of *PtCA1* and *PtCA2*, which contains TGACGT/C motifs at both promoter DNA strands (Ohno et al. 2012). Further, the CO_2_-responsive mechanism to control the accumulation of TpBST1 and TpBST2 at the posttranscriptional levels is still unclear.

Two types of subcellular localization were observed among BST isoforms in *P. tricornutum*. Whereas PtBST1, PtBST2, and PtBST4 were localized in the stroma thylakoid membrane outside of the pyrenoid, PtBST3 was specifically localized in the pyrenoid (Fig. 2, A and B). In *T. pseudonana*, both TpBST1 and TpBST2 were localized at the PPT membrane even though a part of TpBST1 also dispersed to the outside of the pyrenoid (Fig. 2C). We initially assumed that such differences of localization reflected the thylakoid-domain-dependent differences in the function of BSTs; that is, BSTs in the PPT membrane would play a specific role in the CO_2_-evolving machinery but ones in the stroma thylakoid membranes would contribute to more general functions of the photosystems such as regulation of thylakoid-membrane potential by providing a counter anion to the lumen. However, the striking phenotype of ΔPtBST1 mutants, which almost lacks in the LC-inducible CCM, clearly indicates that even the BST factors outside of the pyrenoid are vital components of the CO_2_-evolving machinery in *P. tricornutum*. In addition, the key enzyme for the CO_2_-evolving machinery, Ptθ-CA1, is very specifically localized at the PPT lumen (Kikutani et al. 2016), and the knockout of Ptθ-CA1 completely abolishes not only the LC-inducible CCM but also the constitutive level CCM, clearly indicating the essentiality of Ptθ-CA1 for the CO_2_-evolving machinery in *P. tricornutum* (Shimakawa et al. 2023). Given these considerations, we assume that the stroma thylakoid membranes are highly likely to be connected to the PPT membrane in *P. tricornutum*, and HCO_3_^−^ incorporated into the lumen of the stroma thylakoid membranes can readily move to PPT lumen where it is rapidly dehydrated by Ptθ-CA1 to evolve CO_2_ (Fig. 7). It is unclear so far whether or not the connection between the 2 types of thylakoid membrane is a general feature of diatoms. It is also unclear how the function of PPT-located BST isoforms differs from that of stroma thylakoid-located ones. It is possible that there are some structural variations regarding the relationship between the 2 types of thylakoid membranes among diatom species, which might provide slightly different mechanism of the CO_2_-evolving machinery of the pyrenoid to the case of *T. pseudonana*. It is also possible that, similar to the dynamics of pyrenoid structure and functional alterations observed in Chlamydomonas, the diatom BST isoforms examined in this study might be differentially expressed or localized in response to the severity of CO_2_ starvation, and the PPT-located ones may function in more severe CO_2_ starvation events. Interestingly, GFP-tagged PtBST3 was detected in stroma thylakoid membranes if the C-terminal extension was truncated (Fig. 2A). This implies the existence of a unique sequence in the C-terminal extension of PtBST3 targeting the protein to the pyrenoid, but we could not find any clear natural disorder sequence nor structural moiety such as amphipathic helix, which was previously reported to occur in pyrenoidal β-CAs (PtCA1 and PtCA2) for them to be localized in the pyrenoid (Kitao and Matsuda 2009). A recent study found that BST4 in *C. reinhardtii* has the unique C-terminal region that contains a Rubisco-binding motif and localizes it in the pyrenoid (Adler et al. 2023). Unfortunately, we could not identify any similarity in the C-terminal region between PtBST3 and CrBST4.

The aforementioned second consideration about the functional diversity of BST (i.e. the possibility of BST at stroma thylakoid membranes as a regulator for the photosystem functions) is still the interesting object of this research. BST belongs to a large protein family of anion transporters, which is broadly conserved in bacteria, animals, and plants. It has been reported that several BSTs can transport HCO_3_^−^ (Qu and Hartzell 2008). Transportation of anions across the thylakoid membrane also affects photosynthetic electron transport, especially at stroma thylakoid membranes. During photosynthetic linear electron transport, H^+^ is released in the thylakoid lumen around PSII and at the Q cycle, storing energy as the proton motive force, which is composed of ΔpH and electric field difference (Δψ) across the thylakoid membranes. In principle, both ΔpH and Δψ are the motive force to drive chloroplast ATP synthase. However, ΔpH is also an important factor in the regulation of light utilization and electron transport in the thylakoid membranes. The lumenal acidity activates violaxanthin de-epoxidase and diadinoxanthin de-epoxidase to accumulate zeaxanthin and diatoxanthin, respectively (Jakob et al. 2001), which is essential to induce dissipation of excess light energy at PSII, the so-called NPQ (more specifically qE quenching). Further, the lumen acidification can cause the suppression of electron transport around the cytochrome *b*_6_*f* complex by downregulating the Q cycle. To optimize these regulatory mechanisms on the thylakoid membranes, photosynthetic organisms modulate the balance of ΔpH and Δψ. In the C_3_ plant *Arabidopsis* (*Arabidopsis thaliana*), the K^+^/H^+^ antiporter KEA3 and the BSTs (AtBST1 and AtBST2; often referred to as voltage-gated Cl^−^ channel, VCCN1) function in balancing ΔpH and Δψ (Armbruster et al. 2014; Duan et al. 2016; Herdean et al. 2016). In the case of diatoms, BST isoforms in the stroma thylakoid theoretically have either positive or negative effects on ΔpH because (i) HCO_3_^−^ may increase the proportion of ΔpH relative to Δψ like Cl^−^ in *A. thaliana*; (ii) the spontaneous dehydration of HCO_3_^−^ in the acidic lumen consumes H^+^ to decrease ΔpH; and (iii) the CO_2_-evolving machinery consumes H^+^ in the lumen of PPT, which in turn may draw down the H^+^ in the stromal thylakoid lumen (Fig. 7). LC-grown ΔPtBST1 mutants showed lower NPQ than the LC-grown WT during CO_2_-saturated photosynthesis (Fig. 6A), implying the latter function (ii and/or iii) of BST. However, the functions of BST in photosynthetic light reactions still remain to be investigated in diatoms.

In the present study, the most abundantly expressed BST, PtBST1, was shown to be a bifunctional factor; it sustains the LC-induced level of the CCM in *P. tricornutum*, most probably as a part of the CO_2_-evolving machinery of the pyrenoid. The study also determined the diverse localizations and expression regulations of multiple LC-inducible BST factors in the chloroplast of both *P. tricornutum* and *T. pseudonana*. Future studies on the function and regulation of these BST factors in response to different levels of CO_2_ starvation would provide knowledge on dynamic changes in the mode of the CO_2_-evolving machinery in the pyrenoid-based CCM in diatoms.

## Materials and methods

### Cultures

The marine diatoms *P. tricornutum* Bohlin (UTEX642) and *T. pseudonana* (Hustedt) Hasle et Heimdal (CCMP 1335) were axenically and photoautotrophically cultured in artificial seawater medium with the addition of 0.31% half-strength Guillard's “F” solution (Guillard and Ryther 1962; Guillard 1975) supplemented with 10 nM sodium selenite under continuous light (20°C, 40 *μ*mol photons m^−2^ s^−1^, fluorescent lamp). The cultures were aerated with ambient air (0.04% CO_2_) or 1% CO_2_ gas. For the culture of *T. pseudonana*, the concentration of NaCl was lowered to 270 mM in the medium. The growth of diatoms was evaluated as the increase in optical density at 730 nm. The growth rate was evaluated as *divisions per day* (Wood et al. 2005) using the data points at logarithmic growth phases (0 to 2 and 1.5 to 2.5 d after inoculation in LC and HC growth conditions, respectively).

### Phylogenetic analysis

Amino-acid sequences of BST family proteins were collected from DiatOmicBase (https://www.diatomicsbase.bio.ens.psl.eu/), JGI Genome Portal (https://genome.jgi.doe.gov/portal/), and NCBI (https://www.ncbi.nlm.nih.gov/). For PtBST1, PtBST2, PtBST4, and TpBST1, the correct full-length sequences were determined for the laboratory strains using a SMARTer RACE 5′/3′ kit (Takara, Shiga, Japan). These sequences were aligned by ClustalW, and a conserved domain was defined in reference to the motif of PtBST1. To this motif, the evolutionary history was inferred by using the maximum likelihood method and Le_Gascuel_2008 model (Le and Gascuel 2008). Initial trees for the heuristic search were obtained automatically by applying Neighbor-Join and BioNJ algorithms to a matrix of pairwise distances estimated using the JTT model and then selecting the topology with superior log likelihood value. A discrete Gamma distribution was used to model evolutionary rate differences among sites. All positions containing gaps and missing data in the amino-acid sequence alignment were eliminated. Evolutionary analyses were conducted in MEGA X (Kumar et al. 2018).

### Expression of GFP fusion proteins

The full-length coding regions of *PtBST1*, *PtBST2*, *PtBST3*, *PtBST4*, *TpBST1*, and *TpBST2* were amplified by PCR with the primers shown in Supplementary Table S1. The fragments of *PtBST1* and *PtBST2* were cloned into the *Eco*RI restriction site in pPha-T1/*egfp* vector (Tachibana et al. 2011) using Mighty Mix (Takara). *PtBST3* and *PtBST4* fragments were cloned using Gibson assembly system (New England Biolabs, Ipswich, MA, USA) into the *Eco*RI restriction site in pPha-NR/*egfp* vector that was developed by replacing *fcpA* promoter in pPha-T1/*egfp* vector with nitrate reductase promoter. The resulting plasmid for PtBST3:GFP was linearized by inverse PCR with the primers shown in Supplementary Table S1 and then ligated with Mighty Mix to generate the plasmid for PtBST3^Δ407−519^:GFP. The coding regions of TpBST1 and TpBST2 were cloned with Mighty Mix into the *Eco*RV restriction site in pTpNR/*egfp* vector (Poulsen et al. 2006).

The resulting plasmids were introduced into the *P. tricornutum* or *T. pseudonana* cells by biolistic particle bombardment (PDS-1000/He, Bio-Rad, Hercules, CA, USA) as previously described (Zaslavskaia et al. 2000). Transformants were screened on 1.2% (*w*/*v*) agar medium containing 100 *μ*g mL^−1^ Zeocin (Invitrogen, Waltham, MA, USA) or 100 *μ*g mL^−1^ Nourseothricin (Jena Bioscience, Germany).

### Confocal fluorescence microscopy

The transformant cells grown in liquid medium were collected at the logarithmic growth phase for the confocal fluorescent microscopy with TCS SP8 (Leica, Wetzlar, Germany). Chlorophyll autofluorescence was evaluated from the emission between 600 and 750 nm excited by a 552-nm laser (intensity, 0.15%). GFP was excited by a 488-nm laser (intensity, 4%), and the green fluorescence was detected at 500 to 520 nm (Shimakawa et al. 2022).

### Immunoelectron microscopy

The transformant of *P. tricornutum* that expresses PtBST1 tagged to GFP was fixed by the procedure previously described (Kikutani et al. 2016). Thin sections were cut with an RMC MT-X ultramicrotome (RCM, Tucson, AZ, USA) and mounted on nickel slot grids, followed by an edging step with 1% (*w*/*v*) hydrogen peroxide. After the blocking step, the sections were reacted with polyclonal anti-GFP antibody (AnaSpec, Fremont, CA, USA) diluted 1:500 in 3% (*w*/*v*) BSA in PBS at 25°C overnight. After rinsing with PBS, they were incubated for 60 min at room temperature with a goat anti-rabbit IgG conjugated to 10-nm colloidal gold particles (1:50 diluted in PBS; BBI Solutions, Crumlin, UK). The thin sections were stained with TI blue (Nisshin EM, Aichi, Japan), following washing with distilled water. The sections were observed with a JEM-1011 electron microscope (JEOL, Tokyo, Japan).

### Reverse transcription qPCR

Cells grown under 0.04% and 1% CO_2_ were harvested and frozen in liquid nitrogen at the logarithmic growth phase, which were kept at −80 °C until total RNA and protein extractions as described below. Total RNA was extracted from the frozen cells to prepare cDNA following the method previously reported (Samukawa et al. 2014). Quantitative PCR (qPCR) was performed with GeneAce SYBR qPCR Mix α No ROX (Nippon Gene, Tokyo, Japan) in Thermal Cycler Dice Real Time System II (Takara). *Actin1* genes (*PtActin1* and *TpActin1*) were used as the reference genes. All primers used are shown in Supplementary Table S2.

### Immunoblotting

The cells were disrupted by a UD-201 sonicator (duty, 40; output 0.5; TOMY, Tokyo, Japan) in 50 mM Tris-HCl (pH 6.8) containing 10% (*v*/*v*) glycerol and 1% (*w*/*v*) SDS for 2 min on ice. Unbroken cells were removed by centrifugation at 2,000 × *g* for 5 min, and the resulting supernatant was analyzed by SDS–PAGE. Following electrophoresis, the proteins were blotted onto a polyvinylidene fluoride membrane and subsequently labeled with custom polyclonal antibodies (Japan Bio Serum, Hiroshima, Japan) specific to the target sites of PtBST1 (GDTTGITMDQPHNA), TpBST1 (AGQGQQEYTEENA), and TpBST2 (WRGQGLDKEEQQY). After washing with a phosphate buffer saline containing 0.05% (*v*/*v*) Tween 20, bound antibodies were revealed with a peroxidase-linked secondary anti-rabbit antibody (Promega, Madison, WI, USA) and visualized by chemiluminescence (ImmunoStar Zeta, Wako, Osaka, Japan). rbcS was detected as a control with a rabbit anti-rbcS antiserum against the 35 to 50 regions of the amino-acid sequence in both *P. tricornutum* and *T. pseudonana* (diluted 1:2,000; Sigma, St. Louis, MO, USA).

### Genome editing of *PtBST1*

The nucleotide locations 1014 to 1033 and 1045 to 1064 in *PtBST1* (*Phatr3_J46336*) were chosen as the target pair of single-guide RNA. The PCR fragment including them was prepared from pPt_dual_sgRNAs as the template with the primers (Supplementary Table S3) according to the method previously reported (Nawaly et al. 2020) and then ligated into the BsaI site of pAC-ctCRISPR-Cas9n-2 (Shimakawa et al. 2023). The resulting plasmid vector was introduced into *P. tricornutum* WT cells by bacterial conjugation with *E. coli* S17-1. Transformants were screened on agar plates supplemented 100 *μ*g mL^−1^ Zeocin (Invitrogen, Waltham, MA, USA), and the candidate colonies, which were selected with fragment size assay of target sequence by genomic PCR, were respread twice under HC condition. Direct sequencing was performed to confirm whether or not each colony was monoclone.

### O_2_ measurement

WT and the mutant cells of *P. tricornutum* were harvested at the logarithmic growth phase and resuspended in a DIC-free F/2 artificial seawater freshly prepared. Chlorophyll *a* concentration of the samples was determined in 100% (*v*/*v*) methanol (Jeffrey and Haxo 1968), and the cell samples were applied to oxygen electrode (Hansatech, King's Lynn, UK) at 10 *µ*g chlorophyll *a* mL^−1^ in the DIC-free artificial seawater (pH 8.1). Simultaneous measurement of net O_2_ evolution rate with total DIC concentration in the sample mixture was achieved by the combination of oxygen electrode measurement with GC-FID analysis (GC-8A, Shimadzu, Kyoto, Japan) during a stepwise addition of NaHCO_3_ as previously reported (Kikutani et al. 2016). The photosynthetic parameters were calculated from the plot of O_2_ evolution rate against DIC concentration by curve fitting with the nonlinear least squares method: *P*_max_, maximum net O_2_ evolution rate; and *K*_0.5_, DIC concentration giving a half of *P*_max_.

### Chlorophyll fluorescence analysis

Pulse-modulated excitation was achieved using an LED lamp with a peak emission of 625 nm in a Multi-Color-PAM (Walz, Effeltrich, Germany). Pulse-modulated fluorescence was detected within the range of wavelength limited by RG 665 long-pass and SP 710 short-pass filters. Cells were illuminated with white actinic light from the LED array at 20 °C. The effective quantum yield of PSII, Y(II), was calculated as (*F_m_*ʹ − *F*ʹ)/*F_m_*ʹ: *F_m_*ʹ, maximum fluorescence from light-acclimated cells; *F*ʹ, fluorescence emission from light-acclimated cells. NPQ was calculated as (*F_m_* − *F_m_*ʹ)/*F_m_*ʹ: *F_m_*, maximum fluorescence from dark-acclimated cells. Short saturation flashes (10,000 *µ*mol photons m^−2^ s^−1^, 600 ms) were applied to determine *F_m_* and *F_m_*ʹ.

### Statistical analysis

The results were analyzed statistically by Student's *t*-test (*P < 0.05).

### Accession numbers

The PtBST1, PtBST2, PtBST4, and TpBST1 coding sequences identified in this study are available in DDBJ/EMBL/GenBank under accession numbers LC790448, LC790449, LC790450, and LC790451, respectively. Accession numbers of the genes used for phylogenetic analyses are shown in Supplementary Table S4.

## Supplementary Material

kiae137_Supplementary_Data

## Data Availability

The data underlying this article are available in the article and in its online supplementary material.

## Supplementary data

The following materials are available in the online version of this article.

**Supplementary Table S1.** Primers used for the plasmid construction for the expression of GFP-fused proteins.

**Supplementary Table S2.** Primers used for reverse transcription qPCR.

**Supplementary Table S3.** Primers used for the plasmid construction for genome editing by CRISPR-Cas9 nickase.

**Supplementary Table S4.** Accession numbers of the genes for phylogenetic analyses.

**Supplementary Figure S1.** Phylogenetic tree of BST family proteins in photosynthetic organisms inferred by the maximum likelihood method with Le and Gascuel (2008) model.

**Supplementary Figure S2.** Amino-acid sequence alignment between PtBST3 and PtBST5 by ClustalW.

**Supplementary Figure S3.** Primary structures of BST isoforms in *P. tricornutum* and *T. pseudonana*.

**Supplementary Figure S4.** Genome DNA sequence of the upstream of *PtBST1*, *PtBST2*, *PtBST3*, and *PtBST4* genes in *P. tricornutum*.

**Supplementary Figure S5.** Genome editing by CRISPR-Cas9 nickase to PtBST1 in *P. tricornutum*.

## Dive Curated Terms

The following phenotypic, genotypic, and functional terms are of significance to the work described in this paper:

BST1 Gramene: AT5G65090
BST1 Araport: AT5G65090

SDS Gramene: AT1G14750
SDS Araport: AT1G14750

KEA3 Gramene: AT4G04850
KEA3 Araport: AT4G04850
