# Supplementary material for: Low-CO2-inducible bestrophins outside the pyrenoid sustain high photosynthetic efficacy in diatoms
Source: Plant Physiol. 2024 Mar 13;195(2):1432–45. doi: 10.1093/plphys/kiae137 (PMC11142338; doi:10.1093/plphys/kiae137)
Supplement: kiae137_Supplementary_Data [file kiae137_supplementary_data.zip › Supplementary_Best_20240202.pdf]

Supplementary materials

Supplemental Table S1. Primers used for the plasmid construction for the expression of GFP-fused proteins.

Name	Sequence (5′-3′)
PtBST1:GFP_F	TTGAATTCATGATGCGAAACTTTGCCAG
PtBST1:GFP_R	AAGAATTCAGGAATGGCATTGTGCGGC
PtBST2:GFP_F	GGGAATTCATGACGAACAATTGTACC
PtBST2:GFP_R	CCGAATTCACGAGGAATGTTCAAAAAG
PtBST3:GFP_F	GCCGCCGACGGAACTTACCACTCGTTCCCG
PtBST3:GFP_R	GCTCACCATGATTATAAACGCACGGCCAATCA
PtBST4:GFP_F	CCACTTGTGCGAACGGATGACACTCAGATGGTGCATCTTC
PtBST4:GFP_R	CGCCCTTGCTCACCATGTTGCTAAACTTTGTTCCAGCGG
PtBST3$^{\Delta 407-519}$:GFP_F	ATGGTGAGCAAGGGCGAGGA
PtBST3$^{\Delta 407-519}$:GFP_R	GCTTGTTCTATCAATTGCCATCTG
TpBST1:GFP_F	GGATATCCTCTCGACATTGTTCAA
TpBST1:GFP_R	GCTGCAGCCCCACATCTTACGAGC
TpBST2:GFP_F	GGCGGCCGCCTCTCGACATTGTTCAA
TpBST2:GFP_R	GCTGCAGCATCTGTGCCAAGGGAC

Supplemental Table S2. Primers used for RT-qPCR.

Name	Sequence (5′-3′)
*PtBST1*_F	GCATGATTTCTCCCGAAGGTTTGC
*PtBST1*_R	CAAATAGTGGTCCACTAAGGAATGGCA
*PtBST2*_F	CGAGATGATGGCCGACCAGAATT
*PtBST2*_R	CAAATCCCTCCATGCATTATGGTTATATGT
*PtBST3*_F	ATCTATAAGCACATCGGAG
*PtBST3*_R	CAAACTGGTATGTCAGTCC
*PtBST4*_F	CTAGCTCAGCGGTTTGTGGA
*PtBST4*_R	CCATCCCCCATCAACACCAA
*PtActin1*_F	GATTGTTGCTCCTCCGGAAC
*PtActin1*_R	CACCTCCTACAAACGTTGAAGAAC
*TpBST1*_F	GATCTTCCTTGGCAAACTTGATTC
*TpBST1*_R	CGTCTTCGGTGTATCCGAGAATA
*TpBST2*_F	GATTGTTGCTCCTCCGGAAC
*TpBST2*_R	CACCTCCTACAAACGTTGAAGAAC
*TpActin1*_F	GGTATTGCTGACTCTACGTTCC
*TpActin1*_R	CCGTAATCTCCTTCGACATAC

Supplemental Table S3. Primers used for the plasmid construction for genome editing by CRISPR-Cas9 nickase.

Name	Sequence (5′-3′)
PtBST-KO_F	AGGTCTCATCGAGGGCGGCATTGTCATAAAAGCGTTTAAGAGCTAGAAAT
PtBST-KO_R	GGGTCTCTAAACTCAAATCCTTGGCGCGTTGTCTCGACTTTGAAGGTGTT

Supplemental Table S4. Accession numbers of the genes for phylogenetic analyses.

Species	Name	Accession number	Database
Phaeodactylum tricornutum	PtBST1	Phatr3_J46336	DiatomicBase
Phaeodactylum tricornutum	PtBST2	Phatr3_J26635	DiatomicBase
Phaeodactylum tricornutum	PtBST3	Phatr3_J46366	DiatomicBase
Phaeodactylum tricornutum	PtBST4	Phatr3_J46360	DiatomicBase
Thalassiosira pseudonana	TpBST1	THAPSDRAFT_4819	DiatomicBase
Thalassiosira pseudonana	TpBST2	THAPSDRAFT_4820	DiatomicBase
Pseudo-nitzschia multistriata	PmBST1	0058080	DiatomicBase
Pseudo-nitzschia multistriata	PmBST2	0053880	DiatoimcBase
Pseudo-nitzschia multistriata	PmBST3	0058090	DiatoimcBase
Pseudo-nitzschia multistriata	PmBST4	0072660	DiatoimcBase
Pseudo-nitzschia multistriata	PmBST5	0006190	DiatoimcBase
Fistulifera solaris	FsBST1	18294	JGI
Fistulifera solaris	FsBST2	1108	JGI
Fistulifera solaris	FsBST3	15251	JGI
Fistulifera solaris	FsBST4	12199	JGI
Fistulifera solaris	FsBST5	10388	JGI
Fistulifera solaris	FsBST6	8605	JGI
Fistulifera solaris	FsBST7	3837	JGI
Fistulifera solaris	FsBST8	9622	JGI
Seminavis robusta	SrBST1	6724	JGI
Seminavis robusta	SrBST2	11791	JGI
Seminavis robusta	SrBST3	30832	JGI
Seminavis robusta	SrBST4	7625	JGI
Seminavis robusta	SrBST5	8791	JGI
Seminavis robusta	SrBST6	19486	JGI
Cyclotella cryptica	CcBST1	9774	JGI
Cyclotella cryptica	CcBST2	9773	JGI
Minidiscus variabilis	MvBST1	394773	JGI
Minidiscus variabilis	MvBST2	773817	JGI
Chlamydomonas reinhardtii	CrBST1	Cre16.g662600	JGI
Botryococcus braunii		Bobra.101_2s0110.1.p	JGI
Volvox carteri		Vocar.0039s0076.1.p	JGI
Coccomyxa subellipsoidea		31392	JGI
Dunaliella salina		Dusal.0033s00032.1.p	JGI
Porphyra umbilicalis		OSX80068	NCBI
Galdieria partita		GJQ09267	NCBI

Galdieria sulphuraria	XP_005709103	NCBI
Gracilariopsis chorda	PXF43435	NCBI
Gracilaria domingensis	KAI0559279	NCBI
Ostreococcus lucimarinus	87902	JGI
Physcomitrium patens	Pp3c1_34560V3.2.p	JGI
Marchantia polymorpha	Mapoly0009s0131.1.p	JGI
Gossypium raimondii	Gorai.007G098800.1	JGI
Theobroma cacao	Thecc.01G360500.1.p	JGI
Solanum tuberosum	Soltu.DM.05G019540.1	JGI
Oryza sativa	LOC_Os03g01570.1	JGI
Emiliania huxleyi	XP_005785617	NCBI
Nannochloropsis oceanica	EWM29617	NCBI
Synechocystis sp. PCC 6803	AGF50632	NCBI
Chrysochromulina tobinii	KOO32217	NCBI
Ectocarpus siliculosus	CBN74931	NCBI
Polarella glacialis	CAE8604725	NCBI
Symbiodinium pilosum	CAE7675978	NCBI
Cladocopium goreaui	CAI3984342	NCBI
Guillardia theta	GUITHDRAFT_157312	NCBI
Undaria pinnatifida	4720	JGI
Saccharina japonica	SJ03908	NCBI
Arabidopsis thaliana	At3G61320	JGI
Malus domestica	MD07G1139000	JGI

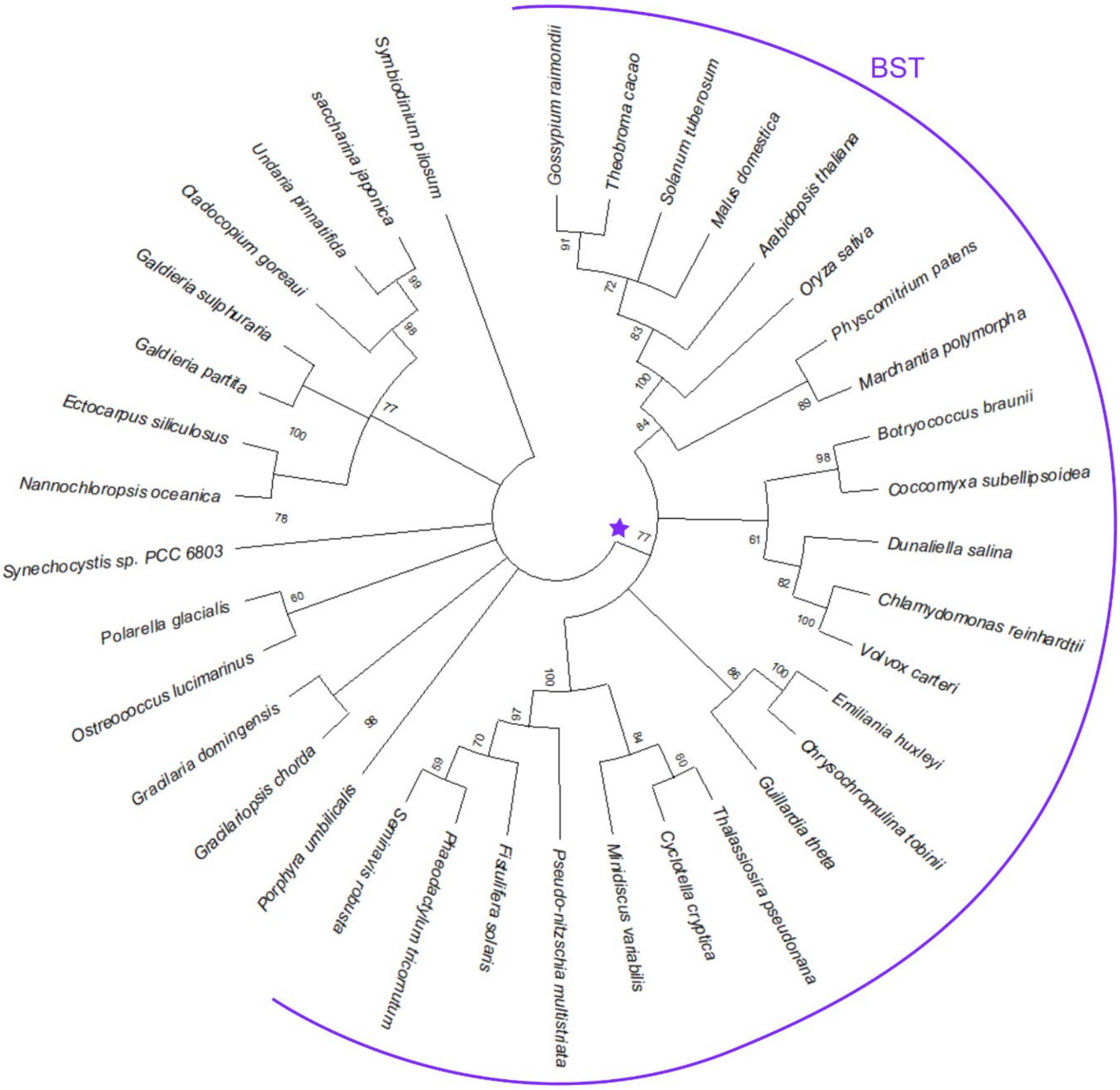

Supplemental Figure S1. Phylogenetic tree of bestrophin family proteins in photosynthetic organisms inferred by the Maximum Likelihood method with Le Gascuel 2008 model. The image was presented as a bootstrap consensus tree in MEGA X. Branches corresponding to partitions reproduced in less than 50% bootstrap replicates are collapsed. Initial tree(s) for the heuristic search were obtained automatically by applying Neighbor-Join and BioNJ algorithms to a matrix of pairwise distances estimated using the JTT model. One each BST ortholog per species showing the highest similarity to PtBST1 were selected for the phylogenetic analysis. Homologs in the BST clade (indicated by purple star) was further analyzed (see the main text).

```
PtBST3          ----MVLQKRLTRGLSFAVILFLASTANKAAAFQASLVSSRTVSRSFDTAIGPVAKDIEP
PtBST5          MRPDCCSFRIGCVMLALASYLGRPAGAFQLSKPIGSSTIGARRSAQPTPTALDVIKVKKP
                        :     *::*  *  .: * : :   .* . .   * .  .:   * *  :*

PtBST3          SPLSYSEKSRLYRRDFYDHDSWLKHRAKNRFVGTLSKVLDSGVVRQLADEVILIGAVATF
PtBST5          DPFSYAEQSRLYRRDFYDHDAWLKHRAKNRFVGTIAKMFESGVIRQLADEIFLIGAVATF
                .*:**:*:************:*************::*:::***:******::********

PtBST3          VCVFNALCVTGYEDFSNLHHDPIFNFGAPLVKLPGEPFSLSSPALSLLLVFKTNTSYQRW
PtBST5          VCLFNSLCVVGYEDFQNVHHDPIFIYGAPLVTLPGEPFSLSSPALSLLLVFKTNTSYQRW
                **:**:***.*****.*:****** :*****.****************************

PtBST3          DEARKAWGVIVNNSRTVIRETSAWVLQSDLSDEEKYRLIRRVADCVWLFPRSLQRHLLNP
PtBST5          DEARKAWGVIVNNSRTVIRESSAWILQTDIPDSEKFRLIHRIAECVWLFPRSLQRHLLNP
                ********************:***:**:*:.*.**:***:*:*:****************

PtBST3          AEDEEAYVKDVRAKLDPVLAEDLVSARHRPTRAMYEMSKAVNTLPLDSYQRSTIDQGVSQ
PtBST5          AEDEEAYVKAVRQRLPPALAEDMVRARHRPTRAMYEMSKAVNTLPLDSIRRSTVDQAVSQ
                ********* ** :* *.****:* *********************** :***:**.***

PtBST3          LCDACGGCERIFGSPVPSIYTRHAARFIELWMFFLPLALYSPFSISWNHWFMIPSSMIIG
PtBST5          LCDACGGCERIFGSPVPSIYTRHAARFLELWIFFLPLALWTPFEFCWNHWFMIPSSMIIG
                ***************************:***:*******::**.:.**************

PtBST3          FFLLGIEELAIQLEEPFSVLPLGKIASGIGLSAEEHVQWAEEQMAIDRTSGPSSYGYPAP
PtBST5          FFLLGIEELAIQLEEPFSVLPLGKIASGIGLSAEEHVQWAEQAMERERTATTDAPPYYR-
                *****************************************: *  :**: ..:  *

PtBST3          IPESTVVSRGPTYSTPTCQLQQTPVPLANGGFSYLNNPSQQSISTSELDNDESSYQEPPT
PtBST5          ------------------------------------------------------------

PtBST3          EQRQPPVVATGDYSYLNPPALSEDGQPQNGGLTYQFGPPRRLP      PtBST3(407-519)
PtBST5          -------------------------------------------
```

Supplemental Figure S2. Amino-acid sequence alignment between PtBST3 and PtBST5 by ClustalW. The C-terminal region of PtBST3 (407−519) is shown in the purple shading.

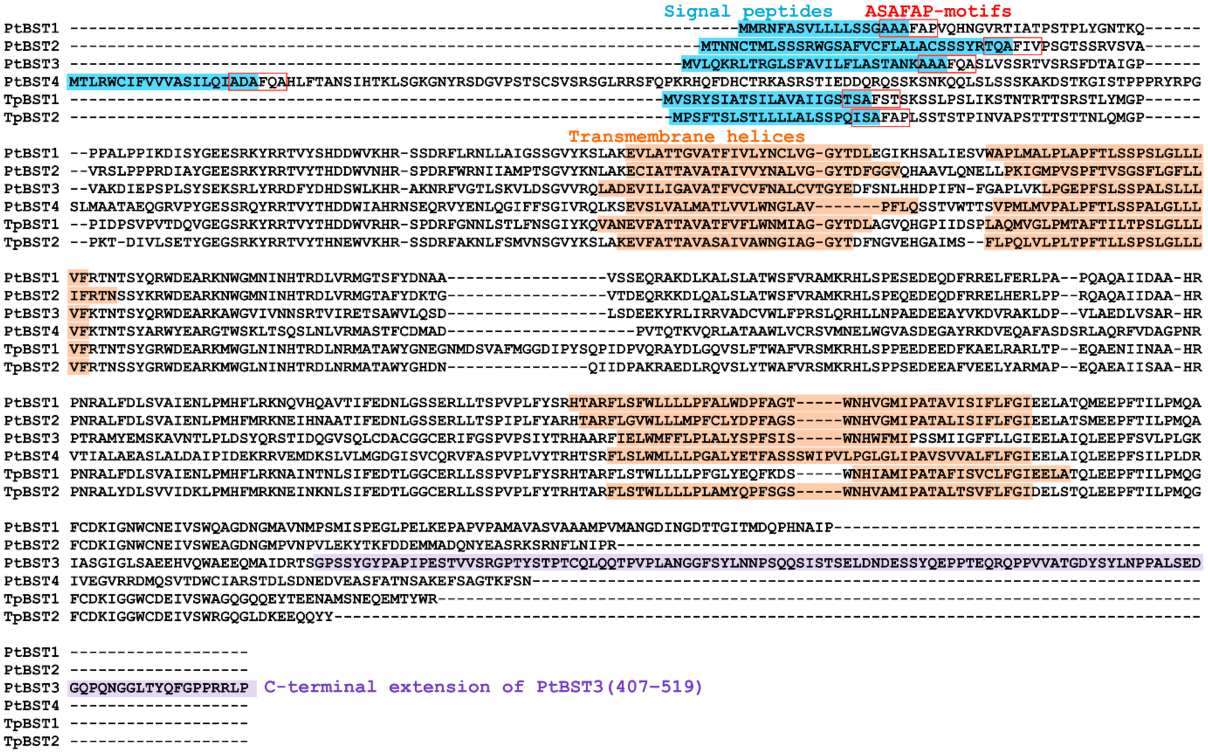

Supplemental Figure S3. Primary structures of BST isoforms in *P. tricornutum* and *T. pseudonana*. The presence of endoplasmic reticulum signal peptide was predicted by SignalP-6.0 (https://services.healthtech.dtu.dk/services/SignalP-6.0/). The plausible ASA-FAP motif for targeting to chloroplasts was manually defined in the region just after the signal peptide. Transmembrane helices were predicted by TMHMM-2.0 (https://services.healthtech.dtu.dk/services/TMHMM-2.0/) according to the transmembrane score >0.1.

>PtBST1
CTTGCGTCACCATTGCGTCAGTTGATAGCCTGACGTCATAGGATGAGTACGACGCGTTCTACGATGGTTTT
ACGTCACAGACTCAGGACACCGGACTTCCTTGTCGAGTATATGCACACGCGGTTTCGAAAAGTGTGTGACG
TGTGCGTTGCGGTTGGGAGAGAAAAGGCCTTTGTATTGTAGATTCTCACAAGTGCGACCTACGTTCTCCGT
TCCGCAGTATCCATTGTTGCAGTCCATTGTCACTACAGTTGTTGCTGTTGCTGTCACTATTATTGTTGTAG
CTACTTTTGTGGAAAAACATGATGCGAAACTTTGCCAGTGTCTTATTGCTGTTGAGCAGCGGCGCGGCTGC
CTTTGCGCCGGTCCAGCACAACGGGGTGCGTACCATCGCCACACCGTCGACGCCGCTCTACGGCAACAC

>PtBST2
CCAAACTTGACCGCCTACTTCACTAAGCTGGTAGGTTATGAAATTCGGATGGGAGTGCCTTCGGCGCGTAC
GTCACAACAGCGTACCCTACCTTTTTGTCACTCTTACGTCAGCACATACGTCACACGTCAGCATCCCCGCC
GAATAGGAATCGGGTCGAGAGTTTGGGTTCTTTCTCACTACCGCGGCGGGGGCCTACTGTTGCGGTACGCA
AATAGCACTGCTATCTCCTACTCGAGAGGGAGAGAAGTACGACGCTCGGATCATCACCCCAACAGCTTTTT
CGACACACAAAGCTCGACCACCATGACGAACAATTGTACCATGTTATCCAGTTCACGTTGGGGATCCGCAT
TCGTCTGTTTTCTCGCGTTGGCGTGTAGCAGTAGCTACCGCACACAGGCGTTCATCGTTCCGTCCGGGACG

>PtBST3
ATGGTGCCCTTGCCCTATGGTTTTAATTCTCTGTAAAGATCGTAGGTAGTTTCTTGAGTGGGCTCTCCAGG
ATCCGAATTTCAGTCATGGATGACGTACGTGCTCGACATGGTGGAAGATGCATACGGTTAATCACCTATGT
ACCTCTTAGCCGATCGGAATACCGGTATTTGTCTGCGTGTCAGTCAAGAGGGGGGACTGACAGTGAGTCTT
CGTTTCGCCCATCAAATCCATCTCATATTGCTGTACTGCTGTTTGTTACATTAGTTACTCGGTTCTTGTCA
CTATTCTCACCAAAAGCATCATGGTGCTACAAAAGCGCCTGACTCGCGGTCTCTCTTTCGCTGTAATTCTT
TTTCTTGCCAGCACTGCGAACAAAGCCGCTGCCTTTCAAGCGTCACTAGTTTCAAGCAGAACCGTATCTCG

>PtBST4
CCAAAATCATCTCCGGGGGGGGGTTTTGGATTGCTGTCCCCTACGCTTGACGGACGGGGACCAAGACGTGA
TGACATCACCGTCCAAAAGCCAATTCGCGAAGATTGAGAGAAGCCGGTAACAACTGGTGAAGATCGATGCC
GTCGCCAAAGAAGCTGCCGAGTCTACTCCTAGATTGAAGCTATAGCTCGTGCTCTGTCTGTTGAGAAATGG
CAAAAGGGGGCATACACTCCTTTACAGAAGACACTCGAAACGGAAATCCACTCAACGTTGCTGCTACCTTC
ACTTGAGATAGTTTACAAATGACACTCAGATGGTGCATCTTCGTTGTAGTGGCCTCCATACTCCAAATCGC
GGATGCCTTCCAAGCACACTTATTCACAGCGAACAGCATTCACACGAAATTGTCAGGAAAGGGGAATTA

Supplemental Figure S4. Genome DNA sequence of the upstream of *PtBST1*, *PtBST2*, *PtBST3*, and *PtBST4* genes in *P. tricornutum*. CO_2-cAMP-responsive elements, termed as CCRE, were identified as indicated by pink and purple boxes. The coding sequences are highlighted in red.

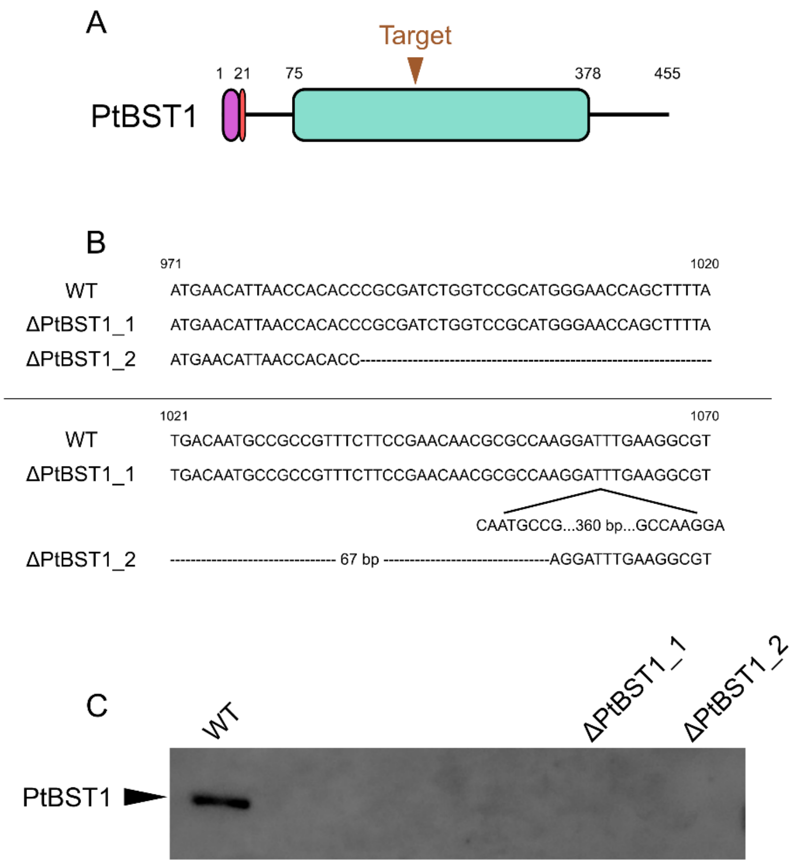

Supplemental Figure S5. Genome editing by CRISPR Cas9 nickase to PtBST1 in *P. tricornutum*. (A) Target site of gRNAs in PtBST1. Blue box shows the conserved motif in bestrophins. Purple and red boxes indicate the signal peptide and ASAFAP motif, respectively. (B) DNA sequence alignment of *PtBST1* in WT and each knock-out mutant. (C) Western blot analysis of WT and knock-out mutants using anti-PtBST1 antibody.
