# Supplementary material for: Low-CO2-inducible bestrophins outside the pyrenoid sustain high photosynthetic efficacy in diatoms
Source: Plant Physiol. 2024 Mar 13;195(2):1432–45. doi: 10.1093/plphys/kiae137 (PMC11142338; doi:10.1093/plphys/kiae137)
Supplement: kiae137_Supplementary_Data [file pp.01568.2023-s03.pdf › pp.01568.2023-s03.pdf]

Plant Physiology®

Low-CO2 inducible bestrophins sustain high photosynthetic efficacy at distant locations from the pyrenoid in diatoms

Minori Nigishi, Ginga Shimakawa, Kansei Yamagishi, Ryosuke Amano, Shun Ito, Yoshinori Tsuji, Chikako Nagasato, and Yusuke Matsuda

Review Timeline:

Submission Date:	09-Oct-2023
Editorial Decision:	18-Nov-2023
Revision Received:	19-Dec-2023
Accepted:	18-Jan-2024

18-Nov-2023

Dr. Yusuke Matsuda
Kansei Gakuin Daigaku Seimei Kankyo Gakubu
Sanda, Hyogo 669-1330
Japan

RE: Low-CO2 inducible bestrophins sustain high photosynthetic efficacy at distant locations from the pyrenoid in diatoms

Dear Dr. Matsuda:

We have received reviews of your manuscript, which you may view at the end of this letter. On the basis of the advice received and my own evaluation, I believe that, pending revision, this article may ultimately be acceptable for publication in Plant Physiology.

When you are ready to submit your revision, click the below link and find the red arrow under your Author Tasks heading for this revision submission.

If you want to visit this version of the manuscript, you may click the link below and then click on the folder that reads "Post Decision Manuscripts".

Link Not Available

When uploading the revised version of this article, please be sure to include as complete an answer as possible to the referees' criticisms during the first round of reviews in the "response to reviewers" section.

Plant Physiology considers a revision for up to 60 days after a manuscript has been provisionally accepted; limited extension of this timeframe is at the discretion of the Monitoring Editor. A reminder notice will be sent to you 2 weeks before the deadline, at which time you may choose to contact the editorial office to request an extension; if so, please provide a justification for your request. After the expiration of this deadline, the article would need to be submitted as a resubmission and would be handled as such.

Thank you very much for giving us an opportunity to review this work. I look forward to receiving the next version.

Sincerely,

Plant Physiology Board of Editors

Want to add this revision deadline to your calendar? Click below!

--- Reviewer comments:
Reviewer #1 (Comments for the Author):

The manuscript by Nigishi et al describes the characterization of the bestrophin family of bicarbonate transporters in model diatoms. By sequence searches, they identify up to five bestrophin proteins in the marine pennate diatom Phaeodactylum tricornutum and two in the marine centric diatom Thalassiosira pseudonana. The sub-plastid localization of PtBST1-3 and TpBST1-2 were further shown by GFP-targeting, with some bestrophin isoforms distributed in stromal thylakoids while others localize exclusively in the pyrenoid-penetrating thylakoids (PPT). All bestrophins accumulated under atmospheric CO2 either through transcriptional activation or through post-transcriptional control. Using CRISPR-mediated genome editing, the authors further examine the function of the stroma lamellae-localized PtBST1 isoform in P. tricornutum. A knockout of BST1 leads to decreased affinity for dissolved inorganic carbon together with an increase in nonphotochemical quenching (NPQ), pointing to a bifunctional role of BST1 in carbon concentration mechanism as well as modulation of NPQ in diatoms. The downregulation of NPQ, according to the authors, arises from the consumption of protons by the bicarbonate dehydration machinery in PPT. This

is an interesting finding. Overall, the presented data are supportive of the conclusions drawn. I have only a few minor comments to make.

1. The authors seem to refer the outer two membranes of diatoms as chloroplast endoplasmic reticulum. This is incorrect. Only the outermost membrane is called as such. The membrane below it is the plasma membrane of the red alga.
2. Line 62, "mechanism how" => "mechanism of how"
3. Line 63, "reason of ecological successfulness of marine diatoms" => "reason for the ecological success of marine diatoms"
4. Please correct "luminal" to "lumenal" throughout the manuscript.
5. Line 155, "inherited to plants" => "inherited by plants"
6. Lines 171-173, please note that the supplementary Fig. S2 shows only amino acid sequence alignment but not DNA sequence. When referring to protein sequence, please use c-terminal and amino acid sequence rather than 3'-extension and coding sequence.
7. Line 273, "mutants showed higher NPQ in inverse relationship". Please rephrase - the meaning is unclear.
8. Lines 298-303, the two sentences are redundant. Please delete one.
9. Line 303-304, I am not sure how are components like PPT constitutively expressed. Here, we are talking about a thylakoid membrane segment but not individual protein or gene. Please use something like "maintained" for PPT.
10. Line 308, "that is an absolutely required to" => "that is absolutely required to"
11. Fig. 4E y-axis shows a major tick interval of 500 mM but the text (lines 324-325) mentions a K0.5 change of 0.5 mM. Isn't the 4E plot scale then off? Please check.
12. Lines 327-328. Please rewrite as something like "much higher than that of the θ-CA PPT mutant of P. tricornutum."
13. Lines 341-342, the meaning is unclear. Please rephrase.
14. Line 350. "locus" is confusing with genetic locus. Please use something like "thylakoid domain-dependent".
15. Line 376, "pyrenoidal β-CAs (PtCA1 and PtCA2) to be" => "pyrenoidal β-CAs (PtCA1 and PtCA2) for them to be"
16. Line 404, comma after "ST membranes".

Reviewer #2 (Comments for the Author):

The manuscript Nigishi et al. investigated the location, expression, and function of Bestrophins, a family of conserved Cl− and/or HCO_3^- transporters, in two marine diatoms Phaeodactylum tricornutum (PtBST1−4) and Thalassiosira pseudonana (TpBST1 and 2). They concluded that PtBST1 transport HCO_3^- at the stroma thylakoid membranes and has critical roles in CCM. Additionally, they identified the differential regulated CCM in these two marine diatoms, at the transcriptional or possible translational level. Furthermore, they showed the C-terminal of PtBST3 may control its pyrenoid localization, which is quite interesting.

Bestrophin paralogs were identified in the green alga Chlamydomonas reinhardtii (CrBST1-3) and proposed to deliver HCO_3^-accumulated in the chloroplast stroma to the thylakoid lumen by Mukherjee et al. PNAS, 2019.

This manuscript conducted similar work in two marine diatoms and provided solid work to understand the function of Bestrophins in diatoms, advancing our understanding of Bestrophins family and the regulation of CCM.

The experiments conducted by the authors are with high quality. The manuscript is well written, concise, and easy to understand.

I have some comments, suggestions, and questions below.

Line 248, "with both mutants showing a significantly lower growth rate (Fig. 4B) ". Fig. 4B, only one star on ΔPtBST1_1 but not on ΔPtBST1_2.

I suggest merging Supplementary Fig. S5. (HC data) to Fig. 4 (LC data). Both sets of data can be presented in two-column figures side by side, one for LC and the other for HC. Both are beautiful data. Although the data in Fig. S5 are controls under HC, it can strongly support the conclusion in Fig. 4. It would be easier to compare HC and LC data this way.

Line 268, "This implies that the occurrence of PtBST1 in ST plays a role in suppressing NPQ as long as the CO_2 supply to the Calvin cycle is not a limiting factor for the linear electron flow of photosystems. ". The data is not enough to support the suppressing role of PtBST1in NPQ. The increased NPQ in PtBST1 mutants could be due to inefficient CO_2 fixation.

Fig. 4G, why did mutants have slightly higher Y(II) than WT under low CO_2 growth condition? Have the authors run statistical tests for all panels in Fig. 4?

Line 308, "that is an absolutely required to sustain photosynthesis by maintaining," This sentence may be incomplete.

Line 372, "Interestingly, the location of PtBST3 shifted from the PPT to ST by truncating the C-terminal extension of PtBST3 (Fig. 2A)". Did the C-terminal truncated PtBST3 mutant with altered localization have some growth or CCM defects? If the

transformation was done on WT background, you may not see a phenotype. This was not clearly indicated in the method part or result session.

line 401, "In P. tricornutum, the lack of PtBST1 increased NPQ (Fig. 4F), probably due to the decreased consumption of H+ in the PPT lumen for the dehydration of HCO3– (Kikutani et al., 2016; Shimakawa et al., 2023) ". You need to measure the level of zeaxanthin and LHCSR/PSBS, possibly also the ΔpH component of pmf before reaching this conclusion or hypothesis.

Line 411, "PtBST1 ... also modulates the ΔpH in the entire thylakoidal photoreaction." The authors did not provide evidence for this. This is just their hypothesis.

It would be better if the manuscript could reduce the No. of abbreviations used. When reading the discussion part of the manuscript, I needed to go back to the introduction to search for some uncommon abbreviations 2-3 times. I suggest leaving only these common and frequently used abbreviations but fully spelling out other uncommon and infrequently used abbreviations to help the readers understand the paper.

Reviewer #3 (Comments for the Author):

The current study describes the low-CO2 inducible bestrophin proteins (BSTs) for sustaining photosynthetic efficacy in diatoms. The aim is to understand the physiological function of BSTs, which is currently unknown. The authors have identified the bestrophin orthologs from two model diatoms, Phaeodactylum tricornutum (Pt) and Thalassiosira pseudonana (Tp), and used BST-GFP fusions to characterize their subcellular location by fluorescent microscopy. They have further validated BST expression at the transcript and protein level under high and low CO2 conditions. CRISPR/Cas9 deletion of PtBST1 confirms it has an important role in the CO2-concentrating mechanism in diatoms. Collectively the paper provides a significant advance in our understanding of inorganic carbon acquisition in the oceans. Indicating that bestrophin proteins play a central role in carbon fixation in both green and red plastid algal lineages. The manuscript is generally well-written although grammar and sentence structure could be improved in places. Figures are appropriate. I found a few points which need clarification.
General comments
As a suggestion I think the authors should consider removing the Tp experimental data and focus on the Pt data where they have a complete story. I think this would streamline the manuscript and increase impact. Although the Tp data is of high interest it currently appears as a bolted-on addition that detracts from the complete dataset the authors have for Pt. It also prompts more questions than it answers. This data could be useful for a future publication by the authors once a more complete understanding of Tp BST function is known.
Specific comments
Line 40: In the abstract the authors mention that PtBST4's subcellular location is verified but this is not shown in the results, please modify the abstract to reflect this.
Line 77, 81, 97, 523: Change 'sea water' to seawater
Line 83: is converted > is converted
Line 66-76: To help the reader visualize the chloroplast structure of diatoms it would be good to have a schematic diagram of the chloroplast highlighting the four-layers of the chloroplast membranes, the triplet thylakoids and the pyrenoid. This would be especially useful if combined with Figure 2.
Line 208-212: How about PtBST2, 3, 4? Do they possess TGACGT/C motifs? Especially in Fig 3A, PtBST3, 4 are significantly upregulated under LC conditions, which could be considered a low-CO2-inducible gene.
Line 294-295: Although the study provides strong evidence that BSTs deliver HCO3- to the PPTs, there are still many unknowns and we think the language of the following sentence should be changed: "In the present study, we identified the molecular mechanism responsible for supplying HCO3– to the CO2-evolving machinery in the PPT lumen as bestrophin." Suggestion: "In the present study, we provide strong evidence that bestrophins supply HCO3– to the CO2-evolving machinery in the PPT lumen."
Line 339-340: PtBest1 > PtBST1
Line 431-433: Are these sequences deposited somewhere accessible?
Table S1. It would be good to include a sequence diagram of the PtBSTs and TpBSTs depicting the ER-signal and chloroplast transit peptides, including the predicted transmembrane domains. This could be an additional supplemental figure or replace Table S1.
Fig 1. It would be helpful to include the type of phylogenetic analysis conducted in the legend, including model and bootstrapping. Currently the number of bootstraps iterations used does not appear to be present in the manuscript.
Further labels to help guide the reader would be useful. For example labelling pennate and centric clades (as described in the main text) on Fig 1B would be useful. Explaining why the red text is used, would also help the reader when comparing between A and B. Alternatively different colours for Pt and Tp labels would make the trees clearer.
Fig 2 legend: PPT > PPTM
It would be helpful to have a cell map for each diatom species or labels pointing to key ultrastuctural details on the confocal images, e.g pyrenoid, thylakoid traversion etc. See comment above.
Fig 4E and Fig S5E y-axis: Is the K0.5 value µM rather than mM?
Fig 4 Graphs are very small - could be enlarged and rearranged to be more visual.

Fig 4A/B - it would be helpful to specify what data points were used in A to calculate the growth rate in B.
Fig 4 H - The model mentioned in the legend is missing from this figure. A model would be very useful and should be included.

Reviewer: 1

The manuscript by Nigishi et al describes the characterization of the bestrophin family of bicarbonate transporters in model diatoms. By sequence searches, they identify up to five bestrophin proteins in the marine pennate diatom Phaeodactylum tricornutum and two in the marine centric diatom Thalassiosira pseudonana. The sub-plastid localization of PtBST1-3 and TpBST1-2 were further shown by GFP-targeting, with some bestrophin isoforms distributed in stromal thylakoids while others localize exclusively in the pyrenoid-penetrating thylakoids (PPT). All bestrophins accumulated under atmospheric CO2 either through transcriptional activation or through post-transcriptional control. Using CRISPR-mediated genome editing, the authors further examine the function of the stroma lamellae-localized PtBST1 isoform in P. tricornutum. A knockout of BST1 leads to decreased affinity for dissolved inorganic carbon together with an increase in nonphotochemical quenching (NPQ), pointing to a bifunctional role of BST1 in carbon concentration mechanism as well as modulation of NPQ in diatoms. The downregulation of NPQ, according to the authors, arises from the consumption of protons by the bicarbonate dehydration machinery in PPT. This is an interesting finding. Overall, the presented data are supportive of the conclusions drawn. I have only a few minor comments to make.

>> Thank you for your helpful comments. We have revised the manuscript in light of the comments:

----

1. The authors seem to refer the outer two membranes of diatoms as chloroplast endoplasmic reticulum. This is incorrect. Only the outermost membrane is called as such. The membrane below it is the plasma membrane of the red alga.

>> We revised the sentence and precisely defined the four membranes (line 59−63).

----

2. Line 62, "mechanism how" => "mechanism of how"

>> We omitted this paragraph because we realized that the Significant statement section is not needed for the journal *Plant Physiology*.

----

3. Line 63, "reason of ecological successfulness of marine diatoms" => "reason for the ecological success of marine diatoms"

>> We omitted this paragraph because we realized that the Significant statement section is not needed for the journal *Plant Physiology*.

----

4. Please correct "luminal" to "lumenal" throughout the manuscript.

>> We revised the terms throughout the manuscript.

----

5. Line 155, "inherited to plants" => "inherited by plants"

>> We revised the sentence (line 145).

----

6. Lines 171-173, please note that the supplementary Fig. S2 shows only amino acid sequence alignment but not DNA sequence. When referring to protein sequence, please use c-terminal and amino acid sequence rather than 3'-extension and coding sequence.

>> We revised the sentence (line 161-162).

----

7. Line 273, "mutants showed higher NPQ in inverse relationship". Please rephrase - the meaning is unclear.

>> We revised the sentence (line 263-269).

----

8. Lines 298-303, the two sentences are redundant. Please delete one.

>> We deleted the sentence (line 288-294).

----

9. Line 303-304, I am not sure how are components like PPT constitutively expressed. Here, we are talking about a thylakoid membrane segment but not individual protein or gene. Please use something like "maintained" for PPT.

>> We revised the sentence (line 295).

----

10. Line 308, "that is an absolutely required to" => "that is absolutely required to"

>> We revised the sentence (line 299).

----

11. Fig. 4E y-axis shows a major tick interval of 500 mM but the text (lines 324-325) mentions a K0.5 change of 0.5 mM. Isn't the 4E plot scale then off? Please check.

>> We corrected the y-axis of the figure.

----

12. Lines 327-328. Please rewrite as something like "much higher than that of the θ-CA PPT

mutant of P. tricornutum."

>> We revised the sentence (line 318−320).

----

13. Lines 341-342, the meaning is unclear. Please rephrase.

>> We revised the sentence (line 332−339).

----

14. Line 350. "locus" is confusing with genetic locus. Please use something like "thylakoid domain-dependent".

>> We revised the sentence (line 345).

----

15. Line 376, "pyrenoidal β-CAs (PtCA1 and PtCA2) to be" => "pyrenoidal β-CAs (PtCA1 and PtCA2) for them to be"

>> We revised the sentence (line 373).

----

16. Line 404, comma after "ST membranes"

>> The text around the indicated sentence was totally revised (line 397−405).

Reviewer: 2

The manuscript Nigishi et al. investigated the location, expression, and function of Bestrophins, a family of conserved Cl− and/or HCO3− transporters, in two marine diatoms Phaeodactylum tricornutum (PtBST1−4) and Thalassiosira pseudonana (TpBST1 and 2). They concluded that PtBST1 transport HCO3− at the stroma thylakoid membranes and has critical roles in CCM. Additionally, they identified the differential regulated CCM in these two marine diatoms, at the transcriptional or possible translational level. Furthermore, they showed the C-terminal of PtBST3 may control its pyrenoid localization, which is quite interesting.
Bestrophin paralogs were identified in the green alga Chlamydomonas reinhardtii (CrBST1-3) and proposed to deliver HCO3−accumulated in the chloroplast stroma to the thylakoid lumen by Mukherjee et al. PNAS, 2019.
This manuscript conducted similar work in two marine diatoms and provided solid work to understand the function of Bestrophins in diatoms, advancing our understanding of Bestrophins family and the regulation of CCM.
The experiments conducted by the authors are with high quality. The manuscript is well written, concise, and easy to understand.
I have some comments, suggestions, and questions below.

>> Thank you for your helpful comments. We have revised the manuscript in light of the comments:

Line 248, "with both mutants showing a significantly lower growth rate (Fig. 4B) ". Fig. 4B, only one star on ΔPtBST1_1 but not on ΔPtBST1_2.

>> We revised the sentences based on the result of the statistical test (line 242).

I suggest merging Supplementary Fig. S5. (HC data) to Fig. 4 (LC data). Both sets of data can be presented in two-column figures side by side, one for LC and the other for HC. Both are beautiful data. Although the data in Fig. S5 are controls under HC, it can strongly support the conclusion in Fig. 4. It would be easier to compare HC and LC data this way.

>> We totally changed the construction of figures in the revised manuscript. Finally, Fig. S5 and Fig. 4 were rearranged into new Figs. 4, 5, and 6.

Line 268, "This implies that the occurrence of PtBST1 in ST plays a role in suppressing NPQ as long as the CO2 supply to the Calvin cycle is not a limiting factor for the linear electron flow of photosystems. ". The data is not enough to support the suppressing role of PtBST1in NPQ. The increased NPQ in PtBST1 mutants could be due to inefficient CO2 fixation.

>> We removed the sentence and revised the part around that (line 263-269). We do not agree that the higher NPQ in the mutants is due to inefficient CO2 fixation because O2 evolution rate and Y(II) are similar between WT and the mutants at saturated CO2 and NPQ was also measured in the same condition.

Fig. 4G, why did mutants have slightly higher Y(II) than WT under low CO2 growth condition? Have the authors run statistical tests for all panels in Fig. 4?

>> We have done statistical tests in the revised manuscript for Y(II) and NPQ between WT and mutants, indicating that the difference was small but significant especially at low light intensities. We do not have any clear interpretations to this (line 263−265).

Line 308, "that is an absolutely required to sustain photosynthesis by maintaining," This sentence may be incomplete.

>> We revised the sentence (line 299−300).

Line 372, "Interestingly, the location of PtBST3 shifted from the PPT to ST by truncating the C-terminal extension of PtBST3 (Fig. 2A)". Did the C-terminal truncated PtBST3 mutant with altered localization have some growth or CCM defects? If the transformation was done on WT background, you may not see a phenotype. This was not clearly indicated in the method part or result session.

>> In this study, we constructed these GFP mutants on WT background. We revised the sentence to avoid the confusion (line 368−369).

Line 401, "In P. tricornutum, the lack of PtBST1 increased NPQ (Fig. 4F), probably due to the decreased consumption of H+ in the PPT lumen for the dehydration of HCO3− (Kikutani et al., 2016; Shimakawa et al., 2023) ". You need to measure the level of zeaxanthin and LHCSR/PSBS, possibly also the ΔpH component of pmf before reaching this conclusion or hypothesis.

>> We agree with this suggestion, and in the revised manuscript we toned down the hypothesis that BST affects NPQ through the proton gradient regulation, which is still just speculation (line 397−405).

Line 411, "PtBST1 ... also modulates the ΔpH in the entire thylakoidal photoreaction." The authors did not provide evidence for this. This is just their hypothesis.

>> We just removed the indicated text.

It would be better if the manuscript could reduce the No. of abbreviations used. When reading the discussion part of the manuscript, I needed to go back to the introduction to search for some uncommon abbreviations 2-3 times. I suggest leaving only these common and frequently used abbreviations but fully spelling out other uncommon and infrequently used abbreviations to help the readers understand the paper.

>> We decreased the number of abbreviations (e.g., CER, CE, and ST). We kept PPT because it often appears and already used in the latest study (Shimakawa et al., 2023).

Reviewer: 3

The current study describes the low-CO2 inducible bestrophin proteins (BSTs) for sustaining photosynthetic efficacy in diatoms. The aim is to understand the physiological function of BSTs, which is currently unknown. The authors have identified the bestrophin orthologs from two model diatoms, Phaeodactylum tricornutum (Pt) and Thalassiosira pseudonana (Tp), and used BST-GFP fusions to characterize their subcellular location by fluorescent microscopy. They have further validated BST expression at the transcript and protein level under high and low CO2 conditions. CRISPR/Cas9 deletion of PtBST1 confirms it has an important role in the CO2-concentrating mechanism in diatoms. Collectively the paper provides a significant advance in our understanding of inorganic carbon acquisition in the oceans. Indicating that bestrophin proteins play a central role in carbon fixation in both green and red plastid algal lineages. The manuscript is generally well-written although grammar and sentence structure could be improved in places. Figures are appropriate. I found a few points which need clarification.

>> Thank you for your helpful comments. We have revised the manuscript in light of the comments:

General comments

As a suggestion I think the authors should consider removing the Tp experimental data and focus on the Pt data where they have a complete story. I think this would streamline the manuscript and increase impact. Although the Tp data is of high interest it currently appears as a bolted-on addition that detracts from the complete dataset the authors have for Pt. It also prompts more questions than it answers. This data could be useful for a future publication by the authors once a more complete understanding of Tp BST function is known.

>> We deeply appreciate the suggestion. We agree that two lines of context of complete analysis from gene listing through functional analysis in strains would be one ideal option. We considered whether or not we should split the data and try to publish Tp part independently. However, it is also true that listing up of BST genes for localization and expression analysis in two model diatoms are an important comparative part of this study. Difference in the expression control between Pt and Tp are also accepted by reviewers as one of interesting points of this study. We therefore decided to leave the Tp data as they are. We also believe better avoids a large manipulation of the context.

Specific comments

Line 40: In the abstract the authors mention that PtBST4's subcellular location is verified but this is not shown in the results, please modify the abstract to reflect this.

>> The sentence had been incorrect, but fortunately we succeeded to construct the mutant of Pt expressing GFP-tagged PtBST4 at the latest experiment. We added the data and related text to the revised manuscript.

Line 77, 81, 97, 523: Change 'sea water' to seawater

>> We corrected the terms throughout the manuscript.

Line 83: is converted > is converted

>> We revised the sentence (line 74).

Line 66-76: To help the reader visualize the chloroplast structure of diatoms it would be good to have a schematic diagram of the chloroplast highlighting the four-layers of the chloroplast membranes, the triplet thylakoids and the pyrenoid. This would be especially useful if combined with Figure 2.

>> We added the concept figure for this manuscript (Fig. 7), which might be useful also to see Fig. 2. Additionally, we added the simple illustrations to Fig. 2.

Line 208-212: How about PtBST2, 3, 4? Do they possess TGACGT/C motifs? Especially in Fig 3A, PtBST3, 4 are significantly upregulated under LC conditions, which could be considered a low-CO_2-inducible gene.

>> We revised the figure (new Supplementary Fig. S4) to show the existence of TGACG motifs in PtBST2, 3, and 4. Additionally, we added the description for this (line 199−206, line 332−339).

Line 294-295: Although the study provides strong evidence that BSTs deliver HCO_3^- to the PPTs, there are still many unknowns and we think the language of the following sentence should be changed: "In the present study, we identified the molecular mechanism responsible for supplying HCO_3^- to the CO_2-evolving machinery in the PPT lumen as bestrophin." Suggestion: "In the present study, we provide strong evidence that bestrophins supply HCO_3^- to the CO_2-evolving machinery in the PPT lumen."

>> We revised the sentence (line 288−294).

Line 339-340: PtBest1 > PtBST1

>> We revised the sentence.

Line 431-433: Are these sequences deposited somewhere accessible?

>> Thank you for the comment. No, those were not deposited. Now, we submitted the sequences of PtBST1, PtBST2, PtBST4, and TpBST1 that we identified by RACE. All accession numbers are shown in the revised manuscript.

Table S1. It would be good to include a sequence diagram of the PtBSTs and TpBSTs depicting the ER-signal and chloroplast transit peptides, including the predicted transmembrane domains. This could be an additional supplemental figure or replace Table S1.

>> We revised Table S1, and it was finally shown as new Supplementary Fig. S3 in the revised manuscript.

Fig 1. It would be helpful to include the type of phylogenetic analysis conducted in the legend, including model and bootstrapping. Currently the number of bootstraps iterations used does not appear to be present in the manuscript.

Further labels to help guide the reader would be useful. For example labelling pennate and centric clades (as described in the main text) on Fig 1B would be useful. Explaining why the red text is used, would also help the reader when comparing between A and B. Alternatively different colours for Pt and Tp labels would make the trees clearer.

>> We revised Fig. 1 following the helpful suggestions.

Fig 2 legend: PPT > PPTM

It would be helpful to have a cell map for each diatom species or labels pointing to key ultrastuctural details on the confocal images, e.g pyrenoid, thylakoid traversion etc. See comment above.

>> We revised the figure legend and added simple illustrations of diatom cells to Fig. 2.

Fig 4E and Fig S5E y-axis: Is the K0.5 value µM rather than mM?

>> We revised the y-axis of the figures.

Fig 4 Graphs are very small - could be enlarged and rearranged to be more visual.

>> Now, Fig. 4 and S5 were merged to generate new Fig. 4, 5, and 6, which should be more visual than before.

Fig 4A/B - it would be helpful to specify what data points were used in A to calculate the growth rate in B.

>> We calculated the growth rates using the data points from 0 to 2 day after inoculation in LC-grown cells, and from 1.5 to 2.5 days after inoculation in HC-grown cells. We mentioned this also in the manuscript (line 424−426).

Fig 4 H - The model mentioned in the legend is missing from this figure. A model would be very useful and should be included.

>> We added the concept figure as new Fig. 7.

18-Jan-2024
Dr. Yusuke Matsuda
Kansei Gakuin Daigaku Seimei Kankyo Gakubu
Sanda, Hyogo 669-1330
Japan

MSID: PP2023-RA-01568R1
MS TITLE: Low-CO2 inducible bestrophins sustain high photosynthetic efficacy at distant locations from the pyrenoid in diatoms

Dear Dr. Yusuke Matsuda:

I am pleased to inform you that your revised manuscript "Low-CO2 inducible bestrophins sustain high photosynthetic efficacy at distant locations from the pyrenoid in diatoms" has been accepted for publication in Plant Physiology. Your manuscript will be checked for consistency with journal formatting and image standards by one of our Science Editors, who will contact you shortly. They may have a number of queries and suggestions for you. Please keep in mind that they work to increase the online visibility of your article and its attraction for readers from the widest of backgrounds. You will have the opportunity to review any revisions, and I hope you will work constructively with them to get the most out of your research. You will also have the opportunity to review all changes when you receive the author proofs for your article.

Once validated for publication, your article will be sent to a Science Editor for a final review of your manuscript and source files. After your final files are uploaded, your article will appear in the next available issue and the online Preview version of your article will be posted after your signed Copyright and Checklist forms and final source files are received.

Please be sure to include the following in your manuscript file:
Harvard style citations
List of all author contributions and funding information at the beginning of manuscript after the titles and author list.
List all supplemental materials associated with the article, with titles, at the end of the ms, before the references and acknowledgments section.
Complete Figure Legends for all figures appearing in the article listed before the Literature Cited.

Thank you and congratulations on your Plant Physiology paper!

Sincerely,
Plant Physiology Board of Editors

=====
FIRST AUTHOR PROFILES
=====

We recognize the hard work that goes into being First Author of a Plant Physiology paper by publishing a "First Author Feature" for each First Author alongside your paper.

If the first author(s) would like to submit information for a feature on Facebook and Twitter, fill out the form by clicking on the link below:

Plant Physiology First Author Feature Form: https://forms.office.com/r/ZBCGZfhm8F
If you cannot access the form through the link above, please contact Rachel at rbelsky@aspb.org.

To upload an image of yourself to accompany your profile, please put your photo in the correct journal folder at the link below. You must label your image: Author First Name Last Name Article Title.

First Author Profile Image Dropbox: https://www.dropbox.com/scl/fo/xz029p7qjr2jml2rp2kmq/h?dl=0&rlkey=6fkxcaqmxx35ap152b2osy1fz

=====
IMPORTANT REMINDER: PEER REVIEW REPORTS

=====

If you opted to publish a peer review report along with your article during the original submission process, it will be prepared by the editorial staff and publicly posted with your manuscript, inside the zip file that contains any other supplemental material. As a reminder, the peer review report is a public record of all comments from editors and reviewers, as well as your prior responses, as you received them in the decision letters for each draft of your manuscript. If you agreed to publish this report and have changed your mind, or are not sure if you selected this option, please contact the editorial office as soon as possible before signing the license agreement from our publisher.

--- Reviewer comments:
Reviewer #1 (Comments for the Author):

Authors have undertaken the revisions that I requested.

Reviewer #2 (Comments for the Author):

Thanks for replying to my comments! I have no further comments or suggestions.

Reviewer #4 (Comments for the Author):

I thank the authors for their efforts in improving the manuscript, especially the illustrations, which helped visualize the structure surrounding the pyrenoid and the inorganic carbon transport towards Rubisco.
